# Possible Role of CHAD Proteins in Copper Resistance

**DOI:** 10.3390/microorganisms12020409

**Published:** 2024-02-18

**Authors:** Gabriela González-Madrid, Claudio A. Navarro, José Acevedo-López, Luis H. Orellana, Carlos A. Jerez

**Affiliations:** Laboratory of Molecular Microbiology and Biotechnology, Department of Biology, Faculty of Sciences, University of Chile, Santiago 7800003, Chile; gabriela.gonzalez.m@ug.uchile.cl (G.G.-M.); clnavarrol@gmail.com (C.A.N.); jacevedol.1994@gmail.com (J.A.-L.); lhorellana@u.uchile.cl (L.H.O.)

**Keywords:** acidophiles, CHAD proteins, polyphosphates, copper resistance, 3D structure, biomining

## Abstract

Conserved Histidine Alpha-helical Domain (CHAD) proteins attached to the surface of polyphosphate (PolyP) have been studied in some bacteria and one archaeon. However, the activity of CHAD proteins is unknown beyond their interaction with PolyP granules. By using bioinformatic analysis, we report that several species of the biomining acidophilic bacteria contain orthologs of CHAD proteins with high sequence identity. Furthermore, the gene coding for the CHAD protein is in the same genetic context of the enzyme polyphosphate kinase (PPK), which is in charge of PolyP synthesis. Particularly, the group of *ppk* and *CHAD* genes is highly conserved. *Metallosphaera sedula* and other acidophilic archaea used in biomining also contain CHAD proteins. These archaea show high levels of identity in genes coding for a cluster having the same organization. Amongst these genes are *chad* and *ppx*. In general, both biomining bacteria and archaea contain high PolyP levels and are highly resistant to heavy metals. Therefore, the presence of this conserved genetic organization suggests a high relevance for their metabolism. It has been formerly reported that a crystallized CHAD protein contains a copper-binding site. Based on this previous knowledge, in the present report, it was determined that all analyzed CHAD proteins are very conserved at their structural level. In addition, it was found that the lack of YgiF, an *Escherichia coli* CHAD-containing protein, decreases copper resistance in this bacterium. This phenotype was not only complemented by transforming *E. coli* with YgiF but also by expressing CHAD from *Acidithiobacillus ferrooxidans* in it. Interestingly, the strains in which the possible copper-binding sites were mutated were also more metal sensitive. Based on these results, we propose that CHAD proteins are involved in copper resistance in microorganisms. These findings are very interesting and may eventually improve biomining operations in the future.

## 1. Introduction

Inorganic polyphosphates (PolyPs) are linear polymers formed by tens or hundreds of orthophosphate residues joined by energy-rich phospho-anhydrides bonds [1]. This biopolymer is found in the three life domains: bacteria, eukarya and archaea. In bacteria and eukarya, its synthesis is catalyzed by the enzyme polyphosphate kinase (PPK) starting from ATP [2,3]. In crenarchaeota, the enzyme that synthesizes PolyP has not yet been identified [4]. In bacteria and crenarchaea, PolyP is degraded by the exopolyphosphatase enzyme (PPX) which hydrolyzes PolyP, liberating inorganic phosphate (P_i_) [5,6]. 

In bacteria, PolyP is mainly cytoplasmic, and it is possible to see them by using electron microscopy in the form of granules dense to the passing of electrons or in a soluble form [7]. However, not all cell types have the same pattern of PolyP accumulation. These polymers have been related to many phenotypes in bacteria, such as heavy metals resistance and different kinds of stress [8,9,10]. 

Biomining is a strategy using microorganisms to recover important metals such as copper [11,12,13]. Although some heavy metals such as copper and iron are essential for the development of all organisms, they become toxic at high concentrations due to their participation in Fenton-like reactions [14]. Therefore, microorganisms have developed a series of molecular mechanisms that allow them to resist high metal concentrations in their environment [15]. These microorganisms involved in bioleaching are recognized for accumulating significant quantities of inorganic PolyP within their cytoplasm [8,9]. This accumulation contributes to their strong resistance against toxic metals found in their surroundings [8,16].

Tumlirsch and Jendrossek identified a novel class of proteins that are attached to the surface of PolyP granules in some model species of *Alphaproteobacteria*, *Betaproteobacteria*, and *Gammaproteobacteria* [17]. 

These proteins contain a Conserved Histidine Alpha-helical Domain (CHAD). By using their fusion to fluorescent proteins, they observed that these proteins colocalized with PolyP granules. These authors proposed that polypeptides of this family are associated in vivo to PolyP granules. 

CHAD proteins usually have two domains corresponding to CYTH (“CyaB and ThTPase”), which are located at the amino terminal region and a CHAD domain localized at the carboxyl terminal. By fusion of the complete polypeptides or individual domains to fluorescent proteins, it was evidenced that only the CHAD domain is responsible for PolyP binding [17]. Lorenzo-Orts et al. (2019) [18] reported that the CHAD domains can bind PolyP using a central pore and a basic patch covering the “front” and “back” sides of the domain. In spite of CHAD domains having been biochemically characterized as specific and selective PolyP-binding proteins, their biological activity is still unknown. 

Although Lorenzo-Orts et al. (2019) [18] suggested that CHAD domains would not be metal binding domains, in Werten et al. (2019) [19], two different metal-binding sites were proposed in which the predominant metal was cupric ion in PptA, a *Streptomyces chartreusis* CHAD domain-containing protein. Based on this, Werten et al. (2019) [19] suggested that PptA has a role in the metabolism, mobilization, or detection of PolyPs. They also suggested that PptA could act as a sensor or modifier of the intracellular copper concentration while simultaneously determining or affecting PolyP availability [19]. Furthermore, it has been suggested that the CHAD domain of *E. coli* YgiF protein could bind copper ions [20].

Considering CHAD proteins have the capacity to bind PolyP and copper (at least in the case of *S. chartreusis*), we found it of great interest to analyze the possibility that these proteins have a role in copper homeostasis. 

In the present report, by using bioinformatic analysis, it was found that several species of the biomining acidophilic bacteria and archaea contain orthologs of CHAD proteins with high sequence identity. Also, it was determined that all analyzed CHAD proteins are very conserved at their structural level. Furthermore, the genetic context of the gene coding for CHAD proteins from acidophilic microorganisms is conserved and related to PolyP metabolism. It has been previously reported that a crystallized CHAD protein contains a copper-binding site [19]. We found a potential copper-binding site in the *Escherichia coli* CHAD protein (YgiF). In addition, it was found that the lack of YgiF decreases copper resistance in this bacterium. This phenotype was not only complemented by transforming *E. coli* with YgiF but also by expressing CHAD from *Acidithiobacillus ferrooxidans* in it. Interestingly, the strains in which the possible copper-binding sites were mutated became also more sensitive to copper.

The results obtained strongly suggest that CHAD proteins have an important role in copper homeostasis. This could be the connection between the role of PolyP and their dynamics in copper resistance. 

## 2. Materials and Methods 

### 2.1. Bacterial Strains and Culture Media

*E. coli* strains were grown in Luria Bertani (LB) medium containing the appropriate antibiotic when required at 37 °C in an orbital stirrer (150 rpm). The LB medium consisted of tryptone (10 g/L), yeast extract (5 g/L) and NaCl (5 g/L). To select clones of interest, cells were grown in solid plates containing LB-agar (1.5% *w*/*v*) and the required antibiotic. Carbenicillin (100 µg/mL) and chloramphenicol (35 µg/mL) were used. Expressions of genes were induced with 1 mM L-arabinose or 1 mM IPTG (isopropyl-β-D-1-thiogalactopyranoside) in the case of the pKD46 vector or pTrc-His2A vector, respectively.

### 2.2. Construction of ygiF Mutant

*E. coli* mutant strain *ygiF* (hereinafter *ygiF*::cm) was obtained by using the procedure of Datsenko and Wanner [21]. Briefly, PCR primers were designed (ygiF H1P1 and ygiF H2P2, see Table S1) for *E. coli ygiF* gene. *ygiF* codes for inorganic triphosphatase YgiF, which has been identified as a protein from the CHAD family [18,22]. PCR reactions were carried out by using One-Taq polymerase (New England Biolabs™, Ipswich, MA, USA) and the pKD3 vector as template DNA (to amplify chloramphenicol resistance cassette). Thirty cycles were used as well as a 50 °C annealing temperature. For more PCR program details, see Table S2.

Bw25113/pKD46 cells were cultivated at 30 °C in LB medium supplemented with 1 mM L-arabinose and 100 µg/mL of carbenicillin. Cells were then transformed by electroporation with the purified DNA fragment obtained by PCR as already described. 

Shocked cells were added to 1 mL LB medium, incubated 3 h at 37 °C, and then were spread onto 1.5% LB agar supplemented with chloramphenicol to select Cm^R^ transformants. To verify the simultaneous loss of the target fragment (nonmutant) and gain of the new mutant-specific fragment, a PCR reaction was carried out with the flanking locus-specific primers and DNAg from transformed colonies as template. Control colonies were always tested side by side.

### 2.3. Cloning and Expression of CHAD Proteins

Considering the modular nature of some CHAD proteins (CYTH and CHAD domains) [18,22], to further explore the role of YgiF protein in copper resistance, the *ygiF* gene and its individual domains were cloned separately to complement the *ygiF*::cm strain. This was also performed with the CHAD protein gene of the acidophilic bacterium *A. ferrooxidans* (*afe_1874,* GenBank: WP_012536814.1). Genes of interest were cloned in the vector pTrcHis2A (Invitrogen^TM^, Waltham, MA USA), following the manufacturer’s instructions. The same procedure was used to clone the individual domains of protein YgiF. Primers used to clone *ygiF* and its domains, and also *afe 1874*, include a stop codon to prevent the presence of a His-tag in these proteins. These primers are detailed in Table S1.

The obtained clones were analyzed to verify correct orientation and the expected sizes of PCR fragments. This was accomplished by PCR using One-Taq polymerase (New England Biolabs™) and the appropriate primers (Table S1) during 30 cycles and an annealing temperature of 50 °C (for more details of PCR, see Table S2). 

### 2.4. Construction of YgiF Variant Strains

Site-directed mutagenesis was completed by PCR [23] in histidines 345 and 349. These mutations imply changing the sequence of CAU codon coding for histidines by GCU codon coding for alanine (Figure S10). This should result in a loss of function [18]. 

Briefly, mutagenic primers containing in their sequence the changed codons for the *ygiF* gene were designed: ygiFH349Afd, ygiFH349Arv, ygiFH345Afd and ygiFH345Arv (see Table S1). 

Two PCR reactions (fragments 1 and 2) were carried out by using Pfu Ultra II Fusion HS DNA Polymerase (Agilent™, Santa Clara, CA, USA) and *E. coli* DNAg as template by using 30 cycles and a 56 °C annealing temperature. For more PCR program details, see Table S2. An overlap PCR reaction was developed by using the previous reactions as a template. Finally, to amplify the construction containing the mutation of protein YgiF, a new reaction was carried out with the external primers ygiF rve and ygiF fwe (Table S1). To generate the double mutant containing both mutated His345 and His349, the same procedure previously described was used. Mutant versions of YgiF (ygiF*H345A, ygiF*H349A and ygiF*H345,349A) were transformed into strain *ygiF*::cm. Site-directed mutants were verified by Capillary Electrophoresis Sequencing (CES) (Macrogen, Seoul, Republic of Korea) (Figure S10). 

### 2.5. Copper Resistance Assays

To determine whether the absence of the YgiF protein affected copper resistance in *E. coli*, both wild-type (Bw25113) and *ygiF*::cm of *E. coli* strains (transformed with empty pTrc-his2A plasmid) were grown, and when they reached 0.1 at O.D._600_, about 3 or 3.5 mM copper sulfate were added, considering the copper minimum inhibitory concentration (MIC value) [24]. This point was considered time 0. Liquid cultures were kept at 37 °C with stirring, and O.D._600_ was measured every 1 h during 8 h after being exposed to the metal. The same procedure was carried out for the mutant strains complemented with *ygiF::cm*/YgiF; *ygiF::cm*/Afe (*ygiF::cm* expressing Afe_1874 from *A. ferrooxidans*); *ygiF::cm*/dCHAD (*ygiF::cm* expressing CHAD domain from YgiF) and *ygiF::cm*/dCYTH (*ygiF::cm* expressing CYTH domain from YgiF). The experiments were performed as three biological replicates—that is, on separate days (different independent cultures).

### 2.6. Bioinformatics Analysis

The search for orthologs of CHAD domain-containing protein from *Acidithiobacillus ferrooxidans* and from *Saccharolobus solfataricus* (SsCHAD, [18]) was conducted by using a Position-Specific Iterative (PSI)-BLAST analysis from NCBI and the database RefSeq [25]. Research parameters (general and scoring) used in this search were configured with default settings.

The CHAD protein of *A. ferrooxidans* (GenBank: MDD2746049.1) was used as a reference for the genera: *Acidithiobacillus* (taxid:119977), *Sulfobacillus* (taxid:28033) and *Thiobacillus* (taxid:919). All these microorganisms are known to form part of biomining consortia. In the case of the SsCHAD (Uniprot ID: Q97YW1) protein, the following archaea were used: *Sulfolobus acidocaldarius* (taxid: 2285), *Sa. solfataricus* (taxid: 2287), *Sulfolobus islandicus* (taxid: 43080), *Sulfolobus metallicus* (taxid: 47303), *Metallosphaera sedula* (taxid: 43687) and *Metallosphaera cuprina* (taxid: 1006005).

All sequences obtained were aligned by using the Constraint-based Multiple Alignment Tool (Cobalt) [26] that allows multiple local alignments, using the default alignment parameters and visualized in Jalview Software (Version 7.1). 

The genetic context of genes encoding CHAD proteins was studied by analyzing groups of homologous genes using clinker, which is a Python-based gene cluster comparison pipeline [27]. The genomes of all used species were downloaded from NCBI, and the genomic contexts of interest (the gene that codes for the CHAD-containing protein and 10 kb flanking on it upstream and downstream) were selected and extracted using Snapgene (in-house sequences). Those in-house sequences were used as input files in the clinker. Multi-record files were treated as gene clusters with multiple loci. Amino acid translations of genes in each cluster were extracted and aligned by using BioPhyton package [28]. By default, global alignments were performed using the BLOSUM62 substitution matrix, a gap open penalty of −10 and extension penalty of −0.5. Clinker generates interactive, to-scale gene cluster comparison figures directly from sequence files.

The genome neighborhood networks (GNNs) for the CHAD protein bacteria of the *Acidithiobacillus* genus and biomining archaea were generated using the EFI-Genome Neighborhood Tool (EFI-GNT; https://efi.igb.illinois.edu/efi-gnt/) (accessed on 9 February 2024) [29,30]. The input was the xgmml file for the CYTH and CHAD domain-containing protein in bacteria (*A. ferrooxidans* (Uniprot: A0A2W1KGF8), *A. ferrivorans* (Uniprot: A0A7T4WBF3)*, A. thiooxidans* (Uniprot: A0A1C2HWG7), *A. ferridurans* (Uniprot: A0A2Z6INW0) and *A. caldus* (Uniprot: A0A060A122)) and the CHAD protein in archaea (*M. sedula* (Uniprot: A0A088E592), *M. prunae* (Uniprot: A0A4D8S3A0)*, M. hakonensis* (Uniprot: A0A2U9IWU4), *M. yellowstonensis* (Uniprot: H2C4Q5), *A. infernus* (Uniprot: A0A6A9QC85), *A. brierleyi* (Uniprot: A0A2U9ICA0) and *S. tokodaii* (Uniprot: A0A832WQX7)). The output was generated using the default parameters.

The crystal structures of CHAD proteins found in the PDB database and those from *A. ferrooxidans* (UniProt ID: A0A2W1KGF8)*, Sa. solfataricus* (UniProt ID: Q97YW1) and *M. sedula* (UniProt ID: A4YFE5) available in AlphaFold were analyzed in the VMD program (Visual Molecular Dynamics) [31]. Structural alignments were completed with software MultiSeq VMD (Version 1.9.4) [32,33], which uses the program STAMP (Structural Alignment of Multiples Proteins) [34] to align proteins. The parameters used for this were: level two for similarity (scanscore) and level two for comparison residues (scanslide). The alignment was performed with the option “Slow scan”. The resulting alignment was colored by root-mean-square distance (RMSD) values. The blue areas indicate structural conservation in those regions of the molecules. Conversely, no correspondence in structural proximities at these points results in red coloring, as indicated by the RMSD coloring.

The prediction of Cu^+1^-binding sites in protein CHAD from *E. coli* (YgiF)*, Sa. solfataricus and M. sedula* was made by using the MIB server online (“Metal Ion-Binding site prediction and docking server” http://bioinfo.cmu.edu.tw/MIB accessed on 9 February 2024) [35]. This system predicts metal-binding sites present in crystallized proteins and allows to see not only interactions at a structural level but also the theoretical model in which the prediction is based. 

### 2.7. Statistical Analysis

Graphs and statistical analyses were conducted using GraphPad Prism 6 (GraphPad Software Inc., San Diego, CA, USA). To compare two or more independent groups, a two-ways Anova test was used, and Tukey’s test was used in case significant differences were present. Significance was established in ns (no significant); *p* ≤ 0.05; where * *p* ≤ 0.05, ** *p* ≤ 0.01; *** *p* ≤ 0.001 and **** *p* ≤ 0.0001.

## 3. Results 

### 3.1. Search for Genes Coding for CHAD Proteins in Genomes of Acidophilic Bacteria

The principal aim of the present report was to find out how biomining microorganisms able to accumulate high amounts of inorganic PolyP in their cytoplasm [8,9] become highly resistant to toxic metals present in their environment [8,16]. Due to the interaction of CHAD proteins with PolyPs [17,18], it is of special interest to analyze the presence of this protein in bioleaching microorganisms. 

A PSI-BLAST analysis was conducted by using the *A. ferrooxidans* CHAD protein to search for orthologues in the *Acidithiobacillus* (taxid:119977), *Sulfobacillus* (taxid:28033) and *Thiobacillus* (taxid:919) genera. All of them are known to form part of microbial consortia involved in biomining and being highly resistant to metals such as copper and cadmium [36]. The results indicated proteins from these genera showed 28–98% identity to the CHAD protein from *A. ferrooxidans*.

The sequences obtained from the PSI-BLAST were aligned and colored by identity percentage. The blue intensity correlates with the degree of identity. It is possible to see highly conserved groups of amino acids in acidophilic bacteria CHAD proteins (Figure S1). Amongst them are *A. ferrooxidans*, *Acidithiobacillus thioxidans*, *Acidithiobacillus albertensis*, *Acidithiobacillus caldus* and *Acidithiobacillus ferrivorans*. 

### 3.2. Search for Genes Coding for CHAD Proteins in the Genomes of Biomining Archaea

This PSI-BLAST analysis was conducted by using SSO_1190 protein from *Sa. solfataricus* P2, which corresponds to a CHAD protein [18]. This analysis was developed by using acidophilic archaeal species such as *S. acidocaldarius*, *Sa. solfataricus*, and *S. islandicus*. Other acidophilic archaea known to participate in biomining processes, such as *S. metallicus*, *M. sedula* and *M. cuprina* were also analyzed. The results indicated that CHAD proteins from this kingdom had 30–82% identity with SSO_1190. It can be seen that in this sequence alignment, there is a high identity amongst the CHAD proteins of acidophilic archaea, including those used in biomining (Figure S2).

### 3.3. Genetic Context of Genes Encoding CHAD Proteins Found in Some Biomining Acidophilic Bacteria

Often, genes involved in biological pathways are grouped in close genomic regions whose comparison can generate relevant information about their function [27,37]. For example, it has been reported that genes coding for CHAD proteins often form groups with genes related to PolyP metabolism [18]. Thus, it has been speculated that the CHAD protein’s function could be associated with PolyP metabolism [19,38,39]. In the present report, the genetic context of genes encoding CHAD protein was analyzed in bacteria involved in bioleaching processes, such as those from *Acidithiobacillus* genera. The genetic region encoding the CHAD protein was selected, and 10 kb flanking each extreme was extracted from *A. ferrooxidans* (GenBank: MDD2746049.1), *A. ferrivorans* (GenBank: SMH64088.1)*, A. Licanantay* (WP_176212044.1), *A. ferridurans* (WP_113525645.1) and *A. caldus* (WP_004873136.1).

**Figure 1:**
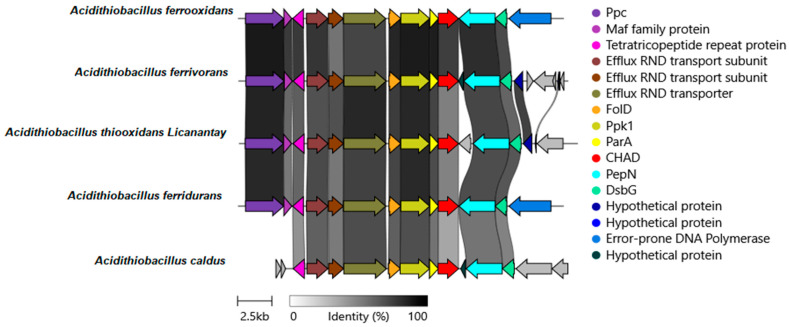
Organization and conservation of CHAD gene clusters in some bioleaching *Acidithiobacillus* reference genomes. Protein names abbreviations used were: Ppc for phosphoenolpyruvate carboxylase, efflux RND transporter for efflux resistance-nodulation-division transporter, FolD for methylenetetrahydrofolate dehydrogenase, Ppk1 for polyphosphate Kinase, ParA for plasmid partition protein A, CHAD for CHAD domain containing protein, PepN to aminopeptidase N, and DsbG to thiol: disulfide interchange protein. The sequence identity percentage is also included. Gene links are shaded based on identity. The scale bar represents 2.5 kb. Image generated with Clinker v0.0.21.

The genetic contexts of each representative microorganism of *Acidithiobacillus* genera were used for the posterior clinker analysis to align the groups of genes and obtain both sequence similarities and degree of conservation. Figure 1 shows the results obtained.

The genomic context of the gene coding for the CHAD protein in all species of *Acidithiobacillus* is very similar. Particularly, the group of *folD, ppk, parA* and *CHAD* genes is highly conserved. FolD is related to NADP metabolism; PPK1 is involved in PolyP metabolism and ParA is crucial in effective plasmids partition in *E. coli.*

In addition, a genome neighborhood analysis was performed using the EFI-GNT server, illustrating the gene encoding the CHAD protein along with potential functions of upstream and downstream genes (Figure S3). No genes related to copper (or other heavy metals) resistance were identified in this genome neighborhood.

### 3.4. Identification of the Genetic Context of Genes Coding for CHAD Proteins in Archaea Involved in Biomining

**Figure 2:**
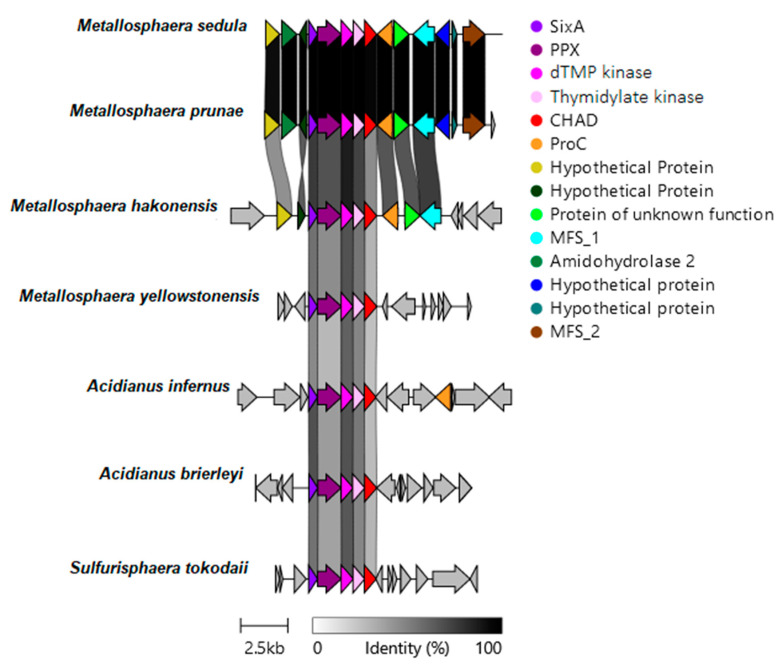
Organization and conservation of the CHAD group of genes in reference genomes from biomining archaea. Protein names abbreviations used were: SixA for phosphohistidine phosphatase, PPX for exopolyphosphatase, dTMP kinase for thymidylate kinase, CHAD for CHAD domain-containing protein, Proc for pyrroline-5-carboxylate reductase, MFS for major facilitator superfamily. Sequence identity percentages are included. Gene links are shaded based on identity. Scale bar represents 2.5 kb. Image generated with Clinker v0.0.21. All the groups of gene sequences were exported from the NCBI database.

The genetic context of genes encoding CHAD proteins was also analyzed in biomining archaea. The genetic flanking regions of CHAD protein genes of representative microorganisms were aligned by using clinker. The obtained results are shown in Figure 2. 

As seen in Figure 2, all analyzed archaea have the same very conserved context around the gene coding for the CHAD protein. Furthermore, close to this genomic environment, the gene coding for PPX is also present.

The genome neighborhoods of these biomining archaea, as well as those in *Acidithiobacillus* bacteria genus, also lack genes related to copper (or other heavy metals) resistance (Figure S4).

### 3.5. Structural Analysis of CHAD Proteins

As already mentioned, CHAD family proteins are associated in vivo to PolyP [17]. On the other hand, it has been reported that under certain stimuli, PolyP granules are rapidly degraded by the activity of PPX. This occurs until PolyP level stabilizes, then stopping its degradation. One of these stimuli is the presence of copper in the culture medium [40].

Copper has a crucial role in the development of cells [15]. This is due to its redox properties, since it acts as a cofactor in numerous enzymes involved in vital functions in cells, such as superoxide dismutases or cytochromes [41]. However, at concentrations higher than the physiological levels, cuprous ions become highly toxic. When free in the cells, its redox cycle makes it extremely toxic, since it catalyzes Fenton-like reactions, generating reactive oxygen species (ROS) [14]. This can cause cellular damage that can be lethal [42]. 

For the above reasons, cells maintain copper homeostasis. They possess different metal resistance mechanisms. In general, these processes are based on (1) inhibition of capturing metals from the medium by diminishing the expression of transporters that enter metals into cells, (2) sequestration of the metal from the exterior of cells or in the cytoplasm by using proteins that bind them, or (3) an active efflux of the toxic ions from cytoplasm to extracellular fraction by means of membrane transporters [43].

Regarding copper-binding proteins, it is widely described that the metal is coordinated mainly by lateral chains of histidine, cysteine, and methionine. The strength of this binding is determined by the combination of these amino acids in the binding site [15]. CHAD proteins are characterized by having conserved domains rich in alpha helices with a high presence of histidine residues. Therefore, it is speculated that these proteins have the capacity to bind divalent cations such as copper by using the mentioned residues [39].

#### 3.5.1. Comparing the General Structure of CHAD Proteins in Biomining Bacteria and Archaea

**Figure 3:**
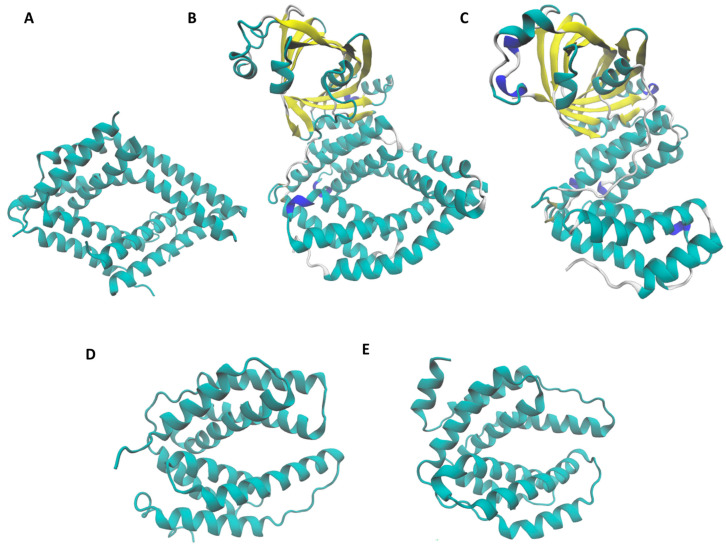
CHAD domains are helical bundles conserved with twofold internal symmetry. (**A**) *S. chartreusis* (PDB ID: 6RN5), (**B**) *A. ferrooxidans* (UniProt ID: A0A2W1KGF8), (**C**) *E. coli* (PDB ID: 5A60), (**D**) *Sa. solfataricus* (UniProt ID: Q97YW1) and (**E**) *M. sedula* (UniProt ID: A4YFE5). C-terminal CHAD domains are in cyan. N-terminal CYTH domains are shown in yellow (**B**,**C**). Blue color indicate zones with low structure prediction accu-racy. Structures reveal the presence of two 4-helix bundles in CHAD domains related by pseudo-twofold symmetry. Note that the CHAD protein from *E. coli* (**C**) contains only one group of 4-helix bundles.

The PDB (https://www.rcsb.org/ accessed on 9 February 2024) database contains CHAD protein structures from *S. chartreusis* (Acces No: 6RN5) and *E. coli* (YgiF protein, Access No: 5A60). In a general way, analyzing the CHAD protein crystals available in the PDB database and the crystal generated by artificial intelligence in the AlphaFold program [44,45], it is striking that all of them share a very similar structure, each containing a CHAD domain rich in alpha-helices (Figure 3). 

CHAD domains conserve a folding of two alpha-helices groups around a central pore. The *E. coli* YgiF protein has a CHAD domain containing only one of the two alpha helices bundles [22]. In addition, *A. ferrooxidans* contains a CYTH domain (in yellow), which has been described in the literature as having adenylate cyclase activity [38].

Using Multiseq analyses, it was possible to observe that as expected, CHAD proteins are well conserved, since they share a high homology at the structural level (Figure S5). The structure alignment was colored by root-mean-square distance (RSMD). The blue areas indicate structural conservation, whereas red ones indicate no correspondence in structural proximities. 

#### 3.5.2. Prediction of YgiF Copper-Binding Sites

**Figure 4:**
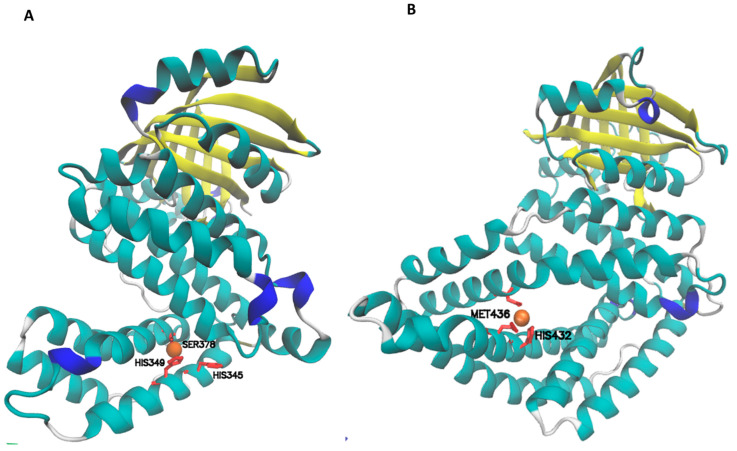
Molecular docking of CHAD protein and Cu^+1^ obtained by using MIB server. (**A**) *E. coli* YgiF protein and Cu^+1^. (**B**) *A. ferrooxidans* CHAD protein and Cu^+1^. Cuprous ions are depicted in orange. The predicted cuprous-binding site is formed by histidine 345 and 349 (in red) to YgiF. Note that VMD also predicts serine 378 residue (in red) as forming part of the binding site for cuprous ions. The predicted cuprous-binding site of *A. ferrooxidans* CHAD protein is formed by histidine 432 and methionine 436 (in red). Yellow color indicates the CYTH domain and blue color indicate zones with low structure prediction accuracy.

Considering *E. coli* is a model Gram-negative microorganism, and many molecular tools are available for its genetic manipulation, it was of interest to perform a docking bioinformatic approach. To find out whether YgiF was capable of binding cuprous ions (normally present in this oxidation state in the cytoplasm), portal MIB was used. This server is capable of modeling and predicting metal-binding sites in proteins having crystallized structures. The result obtained by this server was discharged and visualized by using VMD (Figure 4).

The MIB server identified a possible cuprous-binding site in the CHAD domain of the YgiF protein. The amino acids involved are His345 and His349. This site is in an area very similar to the intramolecular copper-binding site of the CHAD protein from *S. chartreusis*. 

Considering gene contexts are highly conserved and related to PolyP metabolism (Figure 1 and Figure 2) and that protein structures are also highly conserved (Figure S5), CHAD proteins from acidophiles were also analyzed for cuprous ion-binding sites. The result for *A. ferrooxidans* is shown in Figure 4B. The predicted copper-binding site is composed of His 432 and Met 436. Also, this site is near the central pore where PolyP binds [18]. 

Furthermore, CHAD proteins from archaea were analyzed. *M. sedula* and *Sa. solfataricus* CHAD proteins also have possible copper-binding sites. Interestingly, these sites are very similar (Figure S6). 

### 3.6. Activity of CHAD Proteins in Bacteria

Considering CHAD proteins have the capacity to bind PolyP and copper (at least *S. chartreusis* polypeptide), it is interesting to analyze the possibility that these proteins have a role in copper homeostasis. 

#### 3.6.1. Generation of an ygiF Mutant in *E. coli*

Characterization and functional analyses of CHAD proteins were completed in the model bacteria *E*. *coli*. A mutant strain lacking YgiF protein was generated and named *ygiF*::cm using Datsenko and Wanner techniques [21]. 

#### 3.6.2. Metal Resistance Assays

**Figure 5:**
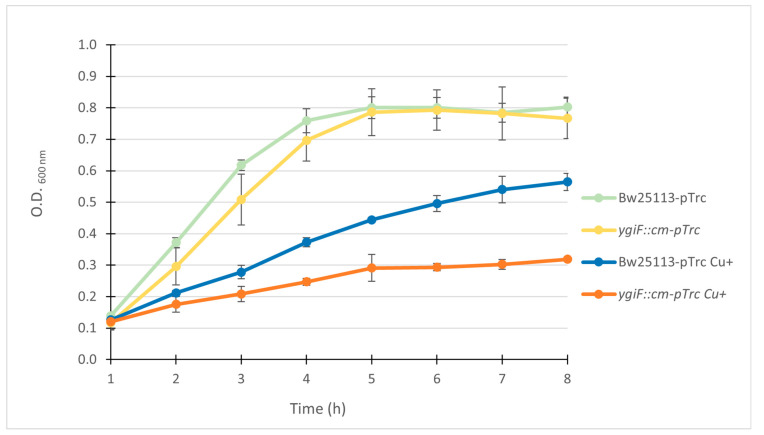
Lack of the ygiF gene increases copper sensitivity in *E. coli.* Copper resistance assays of wild type and *ygiF*::cm *E. coli* mutant (transformed with empty pTrc-his2A plasmid). Cells were grown in LB medium supplemented with 1 mM IPTG in copper absence until they reached an optical density of 0.1. At this point, CuSO_4_ was added to reach 3.5 mM concentration. Next, cultures were incubated at 37 °C, and their respective optical densities were determined at the indicated times. Measurements are the average of three biological replicates. The error bars represent the standard deviations.

It was of interest to determine whether the absence of the YgiF protein affected copper resistance in *E. coli*. For this, liquid cultures of both wild-type (Bw25113) and *ygiF*::cm mutant strains were started in LB medium at 37 °C. When the O.D._600_ measured was 0.1, they were added CuSO_4_ to reach a 3.5 mM concentration. This point was considered time 0. The growth of both strains in the absence and presence of copper was measured for 8 h (Figure 5). 

When strain *ygiF*::cm is exposed to copper, its growth slows down. This demonstrates its higher copper sensitivity compared to the wild-type strain. Starting at 2 h, the differences seen between the two strains are statistically significant, and this difference is higher with the culture time (Figure S7). 

Strains *ygiF*::cm complemented with genes *ygiF* or *Afe_1874* can restore the wild-type phenotype (Figure S8). This supports the idea that the absence of CHAD protein causes a higher sensitivity of strain *ygiF*::cm to copper. 

In contrast, individual domains show significant differences in growth compared with the wild-type strain all the time (Figure S9). This indicates that only a partial restoration is seen. A higher copper resistance was seen in strain *ygiF*::cm when complemented with the CHAD domain as compared with that seen when the CYTH domain was used for complementation (Figure S8).

**Figure 6:**
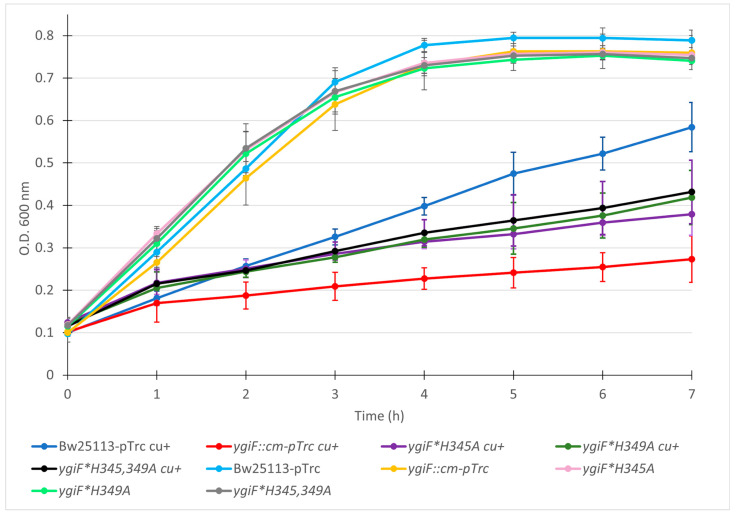
Mutations in the possible copper-binding site of YgiF increase copper sensitivity of *E. coli. E. coli* cells were grown in LB medium supplemented with 1 mM IPTG. When cells reached O.D._600_, 0.1 copper was added to 3.5 mM. Next, cultures were incubated at 37 °C, and O.D._600_ was measured at the indicated times. Measurements correspond to an average of three biological replicates. The error bars represent the standard deviations.

To find out whether the predicted copper-binding site of *E. coli* (Figure 4) has a role in copper homeostasis, site-directed mutagenesis on it was performed. Variant strains ygiF*H345A, ygiF*H349A and ygiF*H345,349A from *E. coli* were evaluated in their sensitivity to copper by using assays for metal resistance (Figure 6).

The variant YgiF strains slow down their growth in a way similar to that of *ygiF::cm* in response to copper. These results suggest that the *E. coli* YgiF protein possibly contains a copper-binding site formed by His345, His349 and Ser378 residues. 

The variant strains showed significant growth differences compared to the wild type one from 4 h onward. On the other hand, strain *ygiF::cm* begins to show significant differences starting at 3 h (Figure S11). 

## 4. Discussion

The activity of CHAD proteins remains unknown beyond their interaction with PolyP granules. In general, both biomining bacteria and archaea contain high PolyP levels and are highly resistant to heavy metals [8,9]. Therefore, within the genetic organization of acidophilic bacteria and biomining archaea (Figure 1 and Figure 2), a cluster of genes related to PolyP metabolism and CHAD-domain-containing proteins is highly conserved. This suggests that CHAD proteins could be involved in PolyP metabolism in acidophilic bacteria and archaea. In addition, Lorenzo-Orts et al. (2019) [18] reported the presence of several CHAD proteins, including those from *Sa. solfataricus, Chlorobium tepidum*, and *Ricinus communis*, in gene clusters encoding PolyP-metabolizing enzymes. The genetic context surrounding the CHAD protein-coding gene (Figure 1 and Figure 2) is not exclusive to biomining organisms.

It has been proposed that a crystallized CHAD protein of *S. chartreusis* (PptA) contains copper-binding sites [19]. Based on this, it has been suggested that PptA may play a role in the detection, mobilization, or metabolism of PolyPs, acting as a potential sensor or modifier of intracellular copper concentration, potentially influencing PolyP availability concurrently [19]. Additionally, the idea that the CHAD domain of the *E. coli* YgiF protein may bind copper ions has been put forth [20]. CHAD proteins might play an important role in copper homeostasis, potentially bridging the functions of PolyP and their dynamics in copper resistance.

Based on this previous knowledge, in the present report, we found a high structural conservation of all analyzed CHAD proteins (Figure 3). Interestingly, the zone where the copper-binding sites of *S. chartreusis* CHAD protein are located is also colored in blue (Figure S5). This strongly suggests these sites could be conserved and may have a role in copper homeostasis. Although this site differs in having a serine residue (Figure 4) instead of histidine, it could also be considered a possible canonical copper-binding site. Furthermore, the high homology level present in the structure of CHAD proteins suggest it is highly conserved in relevant zones such as those seen in the copper-binding site of *S. chartreusis* (Figure S5) [19]. 

We also found that the lack of YgiF decreases copper resistance in *E. coli* (Figure 5). This phenotype was not only complemented by transforming *E. coli* with YgiF but also by expressing CHAD from *A. ferrooxidans* in it (Figure S8). Notably, higher copper resistance was observed in the *ygiF::cm* strain when complemented with the CHAD domain compared to the complementation with CYTH domain (Figure S8). This difference could be due to the fact that only CHAD domain binds to PolyP [17]. Nevertheless, these results suggest that both domains would be necessary to confer copper resistance to *E. coli.* These results (Figure 5) strongly suggest the participation of protein YgiF in the cellular response of *E. coli* to copper. 

Of great interest was finding that those strains in which the possible copper-binding sites were mutated showed a higher sensitivity to copper than wild-type *E. coli* strain (Figure 6). Therefore, although variant strains are more sensitive to copper, they do not reach the same level of copper sensitivity when compared with the *ygiF::cm* strain. This could indicate that although the site has a role in copper resistance provided by protein YgiF, it does not completely explain all metal resistance, and possibly another mechanism also contributes to YgiF copper resistance. 

Based on these results, we propose that CHAD proteins are involved in copper resistance in microorganisms. These findings are of great interest and may eventually have an important role in future biomining operations, especially in microorganisms such as bacteria and archaea thriving in environments with extremely high levels of toxic metals, as encountered in industrial biomining operations for extracting copper and other widely used industrial metals.

## 5. Conclusions

According to our studies, YgiF would be capable of binding Cu^+1^ on its CHAD domain. This site would be composed of His 349, His 345 and Ser 378. In addition, *A. ferrooxidans, Sa. solfataricus* and *M. sedula* would also have putative copper-binding sites formed by His, Cys, and Met. 

By using *E. coli* as a model, the lack of YgiF protein resulted in a strain with higher copper sensitivity compared with the wild type. This phenotype was not only complemented by transforming *ygiF*::cm with YgiF but also by expressing the CHAD protein from *A. ferrooxidans* in it. Furthermore, when the predicted copper ion-binding site in YgiF was mutated, these strains were also more sensitive to the metal.

This suggests the participation of YgiF in the cellular response of *E. coli* to copper. To our knowledge, this is the first evidence for CHAD proteins’ involvement in copper resistance.

Finally, it will be of great interest to further study in more detail the relationship between CHAD proteins, PolyP and copper resistance. In addition, this knowledge could eventually allow improvements in the biomining of copper and other metals.

## Data Availability

No new data were created.

## Supplementary Materials

The following supporting information can be downloaded at https://www.mdpi.com/article/10.3390/microorganisms12020409/s1, Table S1. Primers employed and their uses; Table S2. Details of the PCR program used to generate *E. coli* mutant ygiF::cm; complemented strains YgiF, CHAD, CYTH and Afe; and YgiF variant strains; Figure S1. Alignment of CHAD proteins and consensus sequences from the acidophilic bacteria *Acidithiobacillus* (taxid:119977), *Sulfobacillus* (taxid:28033), and *Thiobacillus* (taxid: 919) genera; Figure S2. Alignment of CHAD protein sequences from some archaea; Figure S3. Genome neighborhood of genes encoding CHAD proteins in bacteria belonging to the *Acidithiobacillus* genus; Figure S4. Genome neighborhood of genes encoding CHAD proteins in biomining archaea; Figure S5. Multiseq analysis of CHAD proteins from *S. chartreusis* (PDB: 6RN5), *E. coli* (PDB: 5A60), *A. ferrooxidans* (UniProt ID: A0A2W1KGF8), *M. sedula* (UniProt ID: A4YFE5), *Sa. solfataricus* (UniProt ID: Q97YW1); Figure S6. Molecular docking for *M. sedula* and *Sa. solfataricus* CHAD proteins and Cu^+1^ obtained by using MIB server; Figure S7. Statistical analysis of copper resistance in the wildtype and *ygiF::cm E. coli* strains; Figure S8. Complemented strains restore wildtype phenotype; Figure S9. Statistical analysis of copper resistance in different *E. coli* mutants; Figure S10. Part of the nucleotide sequences of mutated *ygiF* genes constructed in *E. coli*; Figure S11. Statistical analysis of copper resistance in YgiF variant *E. coli* strains.
