# Supplementary material for: Possible Role of CHAD Proteins in Copper Resistance
Source: Microorganisms. 2024 Feb 18;12(2):409. doi: 10.3390/microorganisms12020409 (PMC10892726; doi:10.3390/microorganisms12020409)
Supplement: Supplementary file 1 [file microorganisms-12-00409-s001.zip › Supplementary Material.pdf]

SUPPLEMENTARY MATERIAL

Table S1. Primers employed and their uses.

Primer	Sequence	Use
ygiF H1P1	AAGTTTATTGTTAATCACAGTGCC GTTGAGGCGTTG CGTG ACCATCTCAATGTAGGCTGGAGC TGCTTCG	To generate *ygiF*::cm mutant strain
ygiF H2P1	AGAACGGTTCCTGATTGTTTGCCTCA TTACGGAAATGTTC AATTTCGATGATGGGAATTAGCCATG GTCC	To generate *ygiF*::cm mutant strain
ygiF fwe	CCATGGCTCAGGAAATCGAATTAAA G	Analyze gene *ygiF* mutation Cloning entire *ygiF* gene
ygiF rve	AAGCTTACGTTTTCCGCTGTGC	Analyze gene *ygiF* mutation Cloning entire *ygiF* gene
ygiF_CH fw	CCATGGGTAAAGCCGATGTGGAACA G	Cloning YgiF CHAD domain
ygiF_CH rv	AAGCTTTTAACGTTTTCCGCTGTGCA	Cloning YgiF CHAD domain
ygiF_CY fw	CCATGGCTCAGGAAATCGAATT	Cloning YgiF CYTH domain
ygiF_CY rv	AAGCTTTTAATTGCCCTGCGCCAGAT	Cloning YgiF CYTH domain
1874_fwNc	CCATGGAACAGGAGCTTAAACTG	Cloning complete gene *afe_1874*
1874_rvHd	AAGCTTGTTCCACGGACGGGGG	Cloning complete gene *afe_1874*
ygFH345Arv	GGCATGGCGGGAAAGAGCGATATCTGCAA AGCGTTTGAAGG	Generation of *ygiF**H345A mutant strain
ygFH345Afd	ACGCTTTGCAGATATCGCTCTTTCCCGCCA TGCC	Generation of *ygiF**H345A mutant strain
ygFH349Arv	CTTTTCAGTTCAGCAGCAGCGCGGGAAAG ATGGATATCGGC	Generation of *ygiF**H349A mutant strain
ygFH349Afd	GCCGATATCCATCTTTCCCGCGCTGCTGCT GAACTGAAAAGC	Generation of *ygiF**H349A mutant strain
ygF*H349rv	GCTTTTCAGTTCAGCAGCAGCGCGGGAAA GAGCGATATCTGC	Generation of *ygiF**H345,349A double mutant
ygF*H349fd	GCAGATATCGCTCTTTCCCGCGCTGCTGCT GAACTGAAAAGC	Generation of *ygiF**H345,349A double mutant

Primers were designed by using SnapGene program version 5.3.1. Thermodynamic properties of primers, such as formation of hairpins, primer dimers and auto aligning sites were analyzed by using "OligoAnalyzer" version 3.1 (www.idtdna.com/analyzer/applications/oligoanalyzer/).

Table S2. Details of the PCR program used to generate *E. coli* mutant *ygiF*::cm; complemented strains YgiF, CHAD, CYTH and Afe; and YgiF variant strains . PCR reactions included 1) denaturation, aligning and 3) extension.

	Step	Cycles number	Temperature (°C)	Duration (min)
PCR 1[a]	1	1	94	2:00
	2	35	94	0:15
			50	0:15
			68	1:30
	3	1	68	5:00
PCR 1.2[b]	1	1	94	5:00
	2	35	94	0:20
			50	0:20
			72	1:30
	3	1	72	5:00
PCR 2[c]	1	1	95	2:00
	2	35	95	0:20
			50	0:20
			72	1:30
	3	1	72	5:00
PCR 1[d]	1	1	95	2:00
	2	35	95	0:20
			56	0:20
			72	1:30
	3	1	72	5:00
PCR 2[e]	1	1	95	2:00
	2	10	95	0:20
			62	0:20
			72	1:30
	3	1	72	5:00

PCR 1.2[f]	1	1	95	2:00
	2	35	95	0:20
			57	0:20
			72	1:30
	3	1	72	5:00

[a] PCR 1: amplification of Wanner fragment comprising a chloramphenicol resistance cassette flanked by homology to gene *ygiF*.

[b] PCR 1.2: confirmation of mutation in *ygiF* gene.

[c] PCR 2: amplification of domain CHAD (YgiF), CYTH (YgiF) and genes *Afe and ygiF*.

[d] Amplification of fragments 1 and 2 of variant strains *ygiF**H345A, *ygiF**H349A and *ygiF**H345,349A

[e] Overlap PCR: fusion of fragments 1 and 2 of *ygiF* mutant gene.

[f] Overlap PCR: amplification of the final mutant fragment.

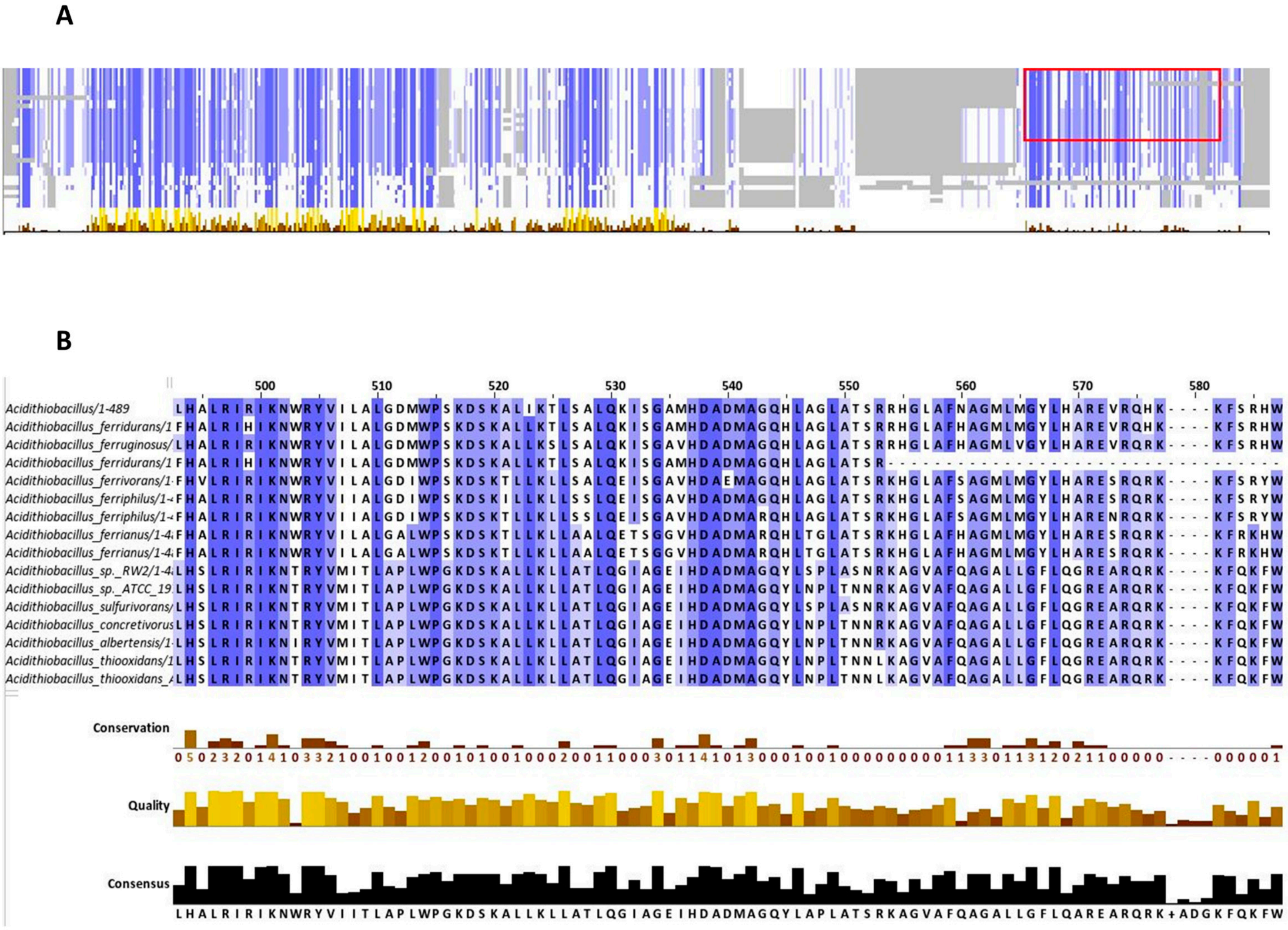

Figure S1. **Alignment of CHAD proteins and consensus sequences from the acidophilic bacteria *Acidithiobacillus* (taxid:119977), *Sulfobacillus* (taxid:28033), and *Thiobacillus* (taxid: 919) genera**. **A.** General view of the chosen sequence alignments containing CHAD proteins. The red rectangle indicates the expanded carboxyl terminal end of CHAD proteins as shown in **B.**

For the alignment, the multiple sequence alignment tool, Cobalt was applied. Alignment result was visualized with Jalview software. The blue intensity correlates with the degree of conservation. Conservation and quality (yellow and brown bars), and consensus sequences (black bars) are also shown.

A

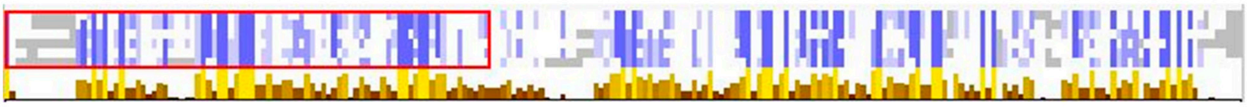

B

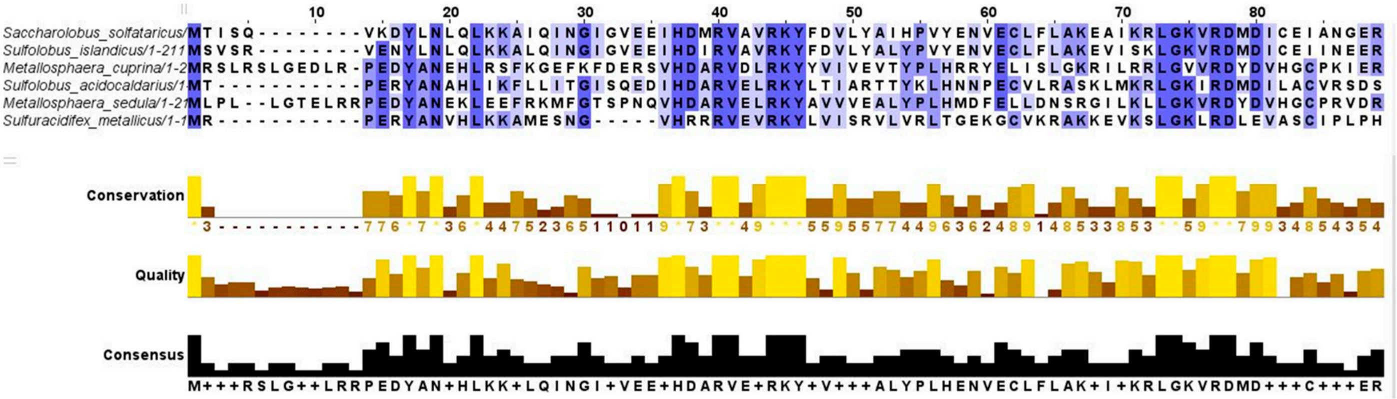

Figure S2. Alignment of CHAD protein sequences from some archaea. *Sa. solfataricus* (taxid: 2287), *S. islandicus* (taxid: 43080), *M. cuprina* (taxid: 1006005), *S. acidocaldarius* (taxid: 2285), *M. sedula* (taxid: 43687) and *S. metallicus* (taxid: 47303) are analyzed. **A.** General view of the chosen sequence alignments containing CHAD proteins.The red rectangle indicates the expanded CHAD proteins as shown in **B.**

For the alignment, the software Multiple Sequence Alignment Clustal Omega was applied. Alignment results are visualized with Jalview software. For a better comparison, an approximation to the carboxyl terminal is shown. This highlights the conserved CHAD domain. The blue intensity correlates with the degree of conservation. Conservation and quality (yellow and brown bars), and consensus sequences (black bars) are also shown.

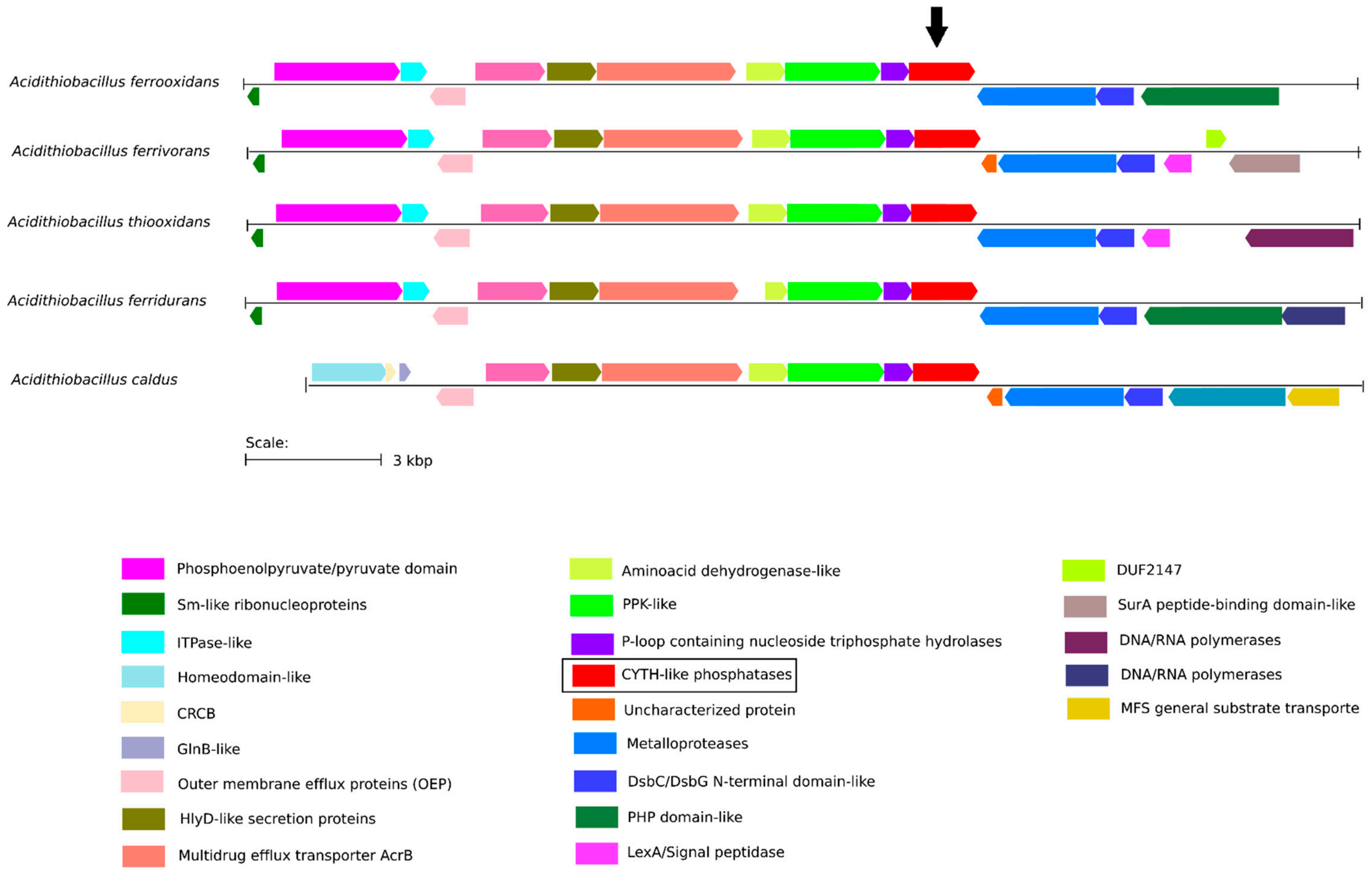

Figure S3. Genome neighborhood of genes encoding CHAD proteins in bacteria belonging to the Acidithiobacillus genus. The genomic neighborhood was generated by using the EFI-Genome Neighborhood Tool (EFI-GNT) which depicts the coding sequence region of CHAD proteins (in red, as indicated with the black arrow) along with putative functions of upstream and downstream genes. The CHAD protein used were: *A. ferrooxidans* (Uniprot: A0A2W1KGF8), *A. ferrivorans* (Uniprot: A0A7T4WBF3), *A. thiooxidans* (Uniprot: A0A1C2HWG7), *A. ferridurans* (Uniprot: A0A2Z6INW0) and *A. caldus* (Uniprot: A0A060A122). Genes with similar functions (either predicted or experimentally determined) are colored the same, based on PFAM superfamilies. Scale bar represents 3 kb. Genes are drawn to scale.

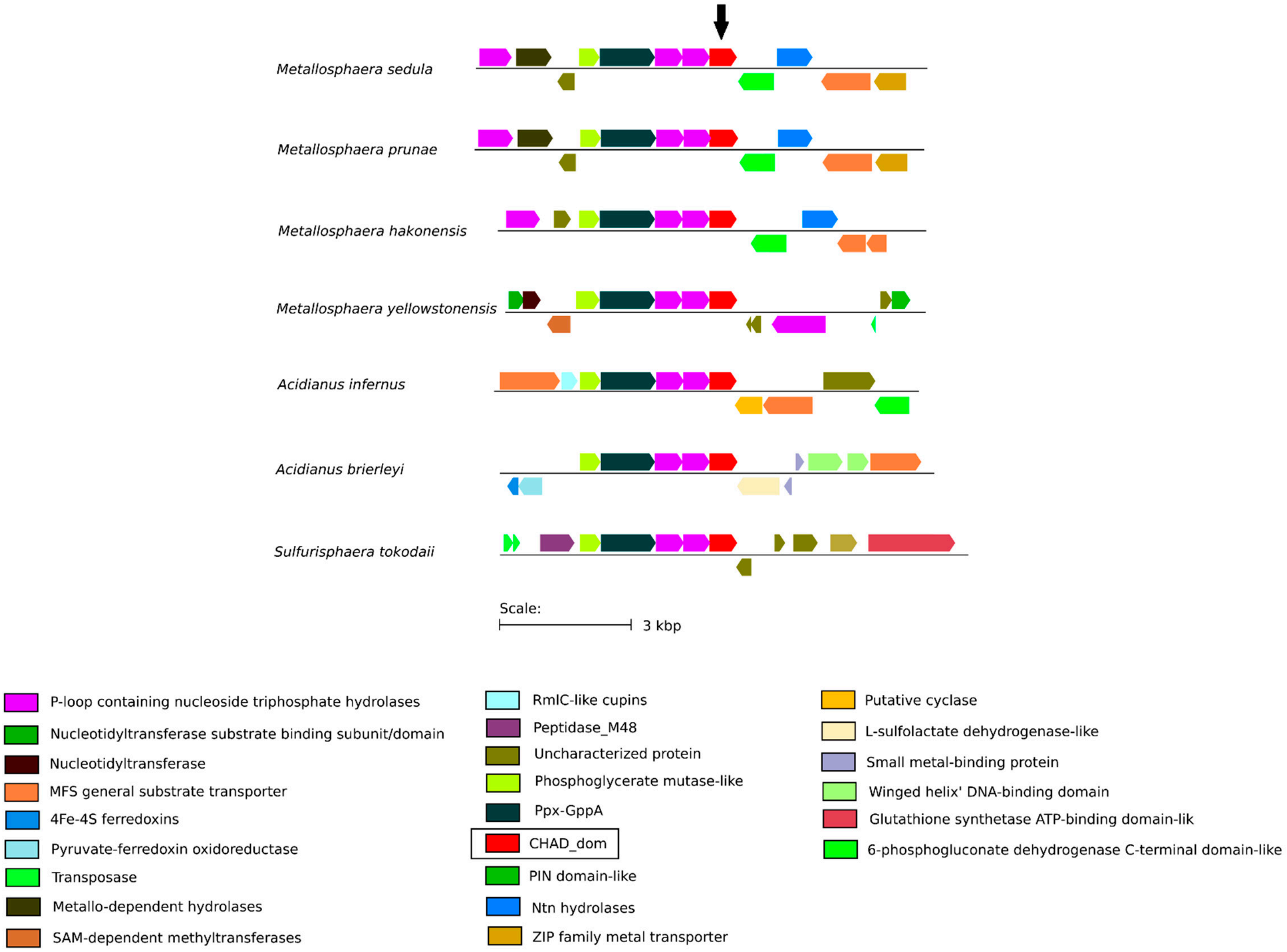

Figure S4. Genome neighborhood of genes encoding CHAD proteins in biomining archaea. The genomic neighborhood was generated by using the EFI-Genome Neighborhood Tool (EFI-GNT) which depicts the coding sequence region of CHAD proteins (in red, as indicated with the black arrow) along with putative functions of upstream and downstream genes. The CHAD protein used were: *M. sedula* (Uniprot: A0A088E592), *M. prunae* (Uniprot: A0A4D8S3A0), *M. hakonensis* (Uniprot: A0A2U9IWU4), *M. yellowstonensis* (Uniprot: H2C4Q5), *A. infernus* (Uniprot: A0A6A9QC85), *A. brierleyi* (Uniprot: A0A2U9ICA0) and *S. tokodaii* (Uniprot: A0A832WQX7). Genes with similar functions (either predicted or experimentally determined) are colored the same, based on PFAM superfamilies. Scale bar represents 3 kb. Genes are drawn to scale.

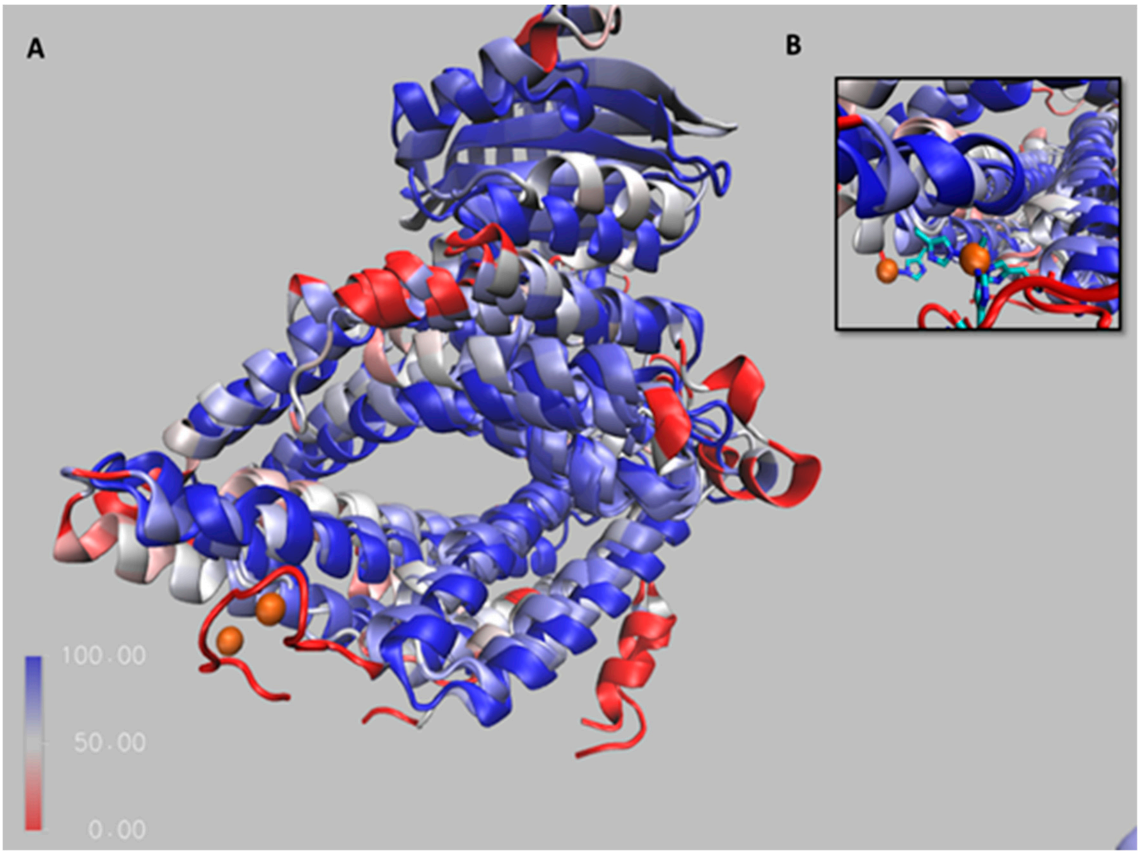

Figure S5. Multiseq analysis of CHAD proteins from *S. chartreusis* (PDB: 6RN5), *E. coli* (PDB: 5A60), *A. ferrooxidans* (UniProt ID: A0A2W1KGF8), *M. sedula* (UniProt ID: A4YFE5), *Sa. solfataricus* (UniProt ID: Q97YW1). A. General view of multiseq analysis. **B.** Zoom on the Cu binding sites of *S. chartreusis* CHAD protein. The structure alignment was coloring by root-mean-square distance (RSMD). The blue areas indicate structural conservation, whereas red areas indicate no correspondence in structural proximities.

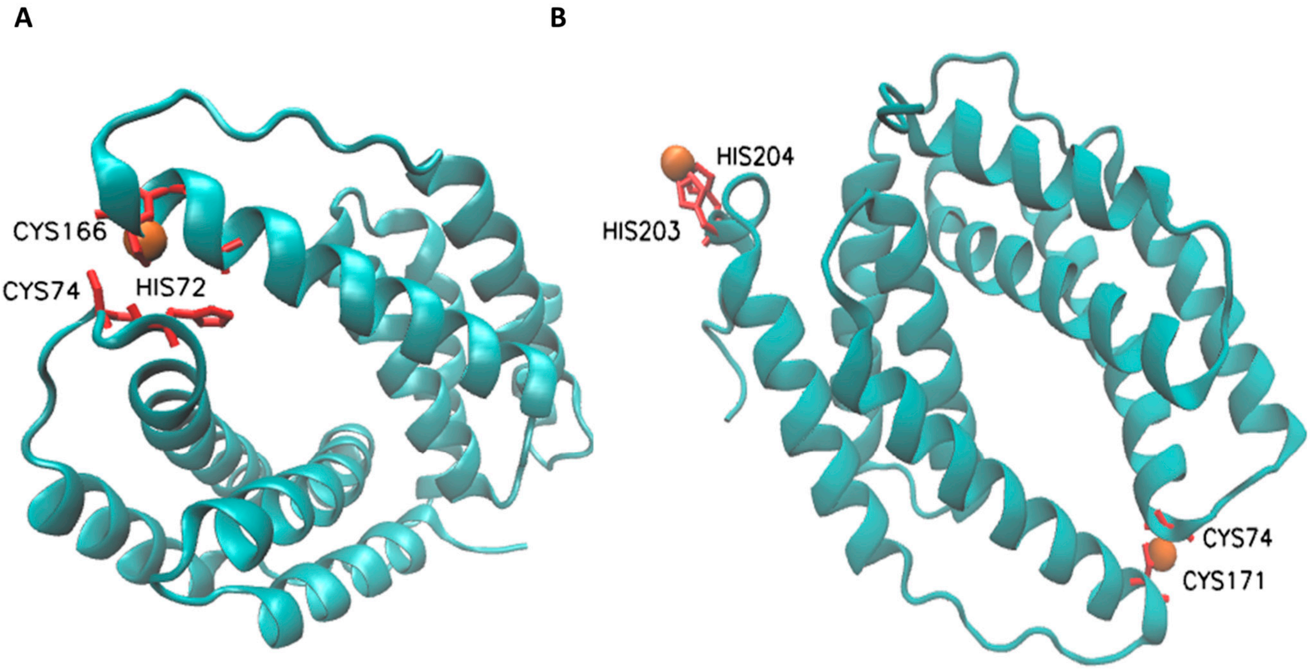

Figure S6. Molecular docking for *M. sedula* and *Sa. solfataricus* CHAD proteins and Cu^{+1} obtained by using MIB server. **A.** Predicted cuprous binding site is formed by His 72, Cys 74, 166 for *M. sedula*. **B.** Two copper binding sites are shown for *Sa. solfataricus* formed by His 203, 204, and Cys 74, 171. Cu^{+1} ion is depicted in orange.

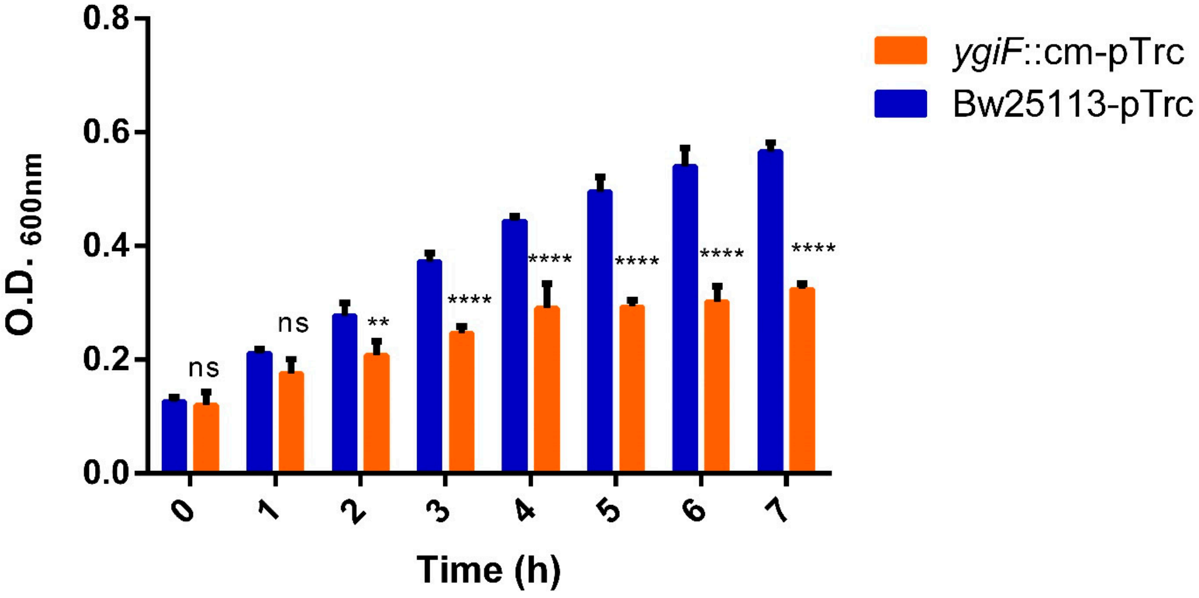

Figure S7. Statistical analysis of copper resistance in the wildtype and *ygiF*::cm *E. coli* strains. Two-way ANOVA test was used, where **** indicates $p \leq 0.0001$, *** $p \leq 0.001$, ** $p \leq 0.01$, * $p \leq 0.05$ and ns not significant.

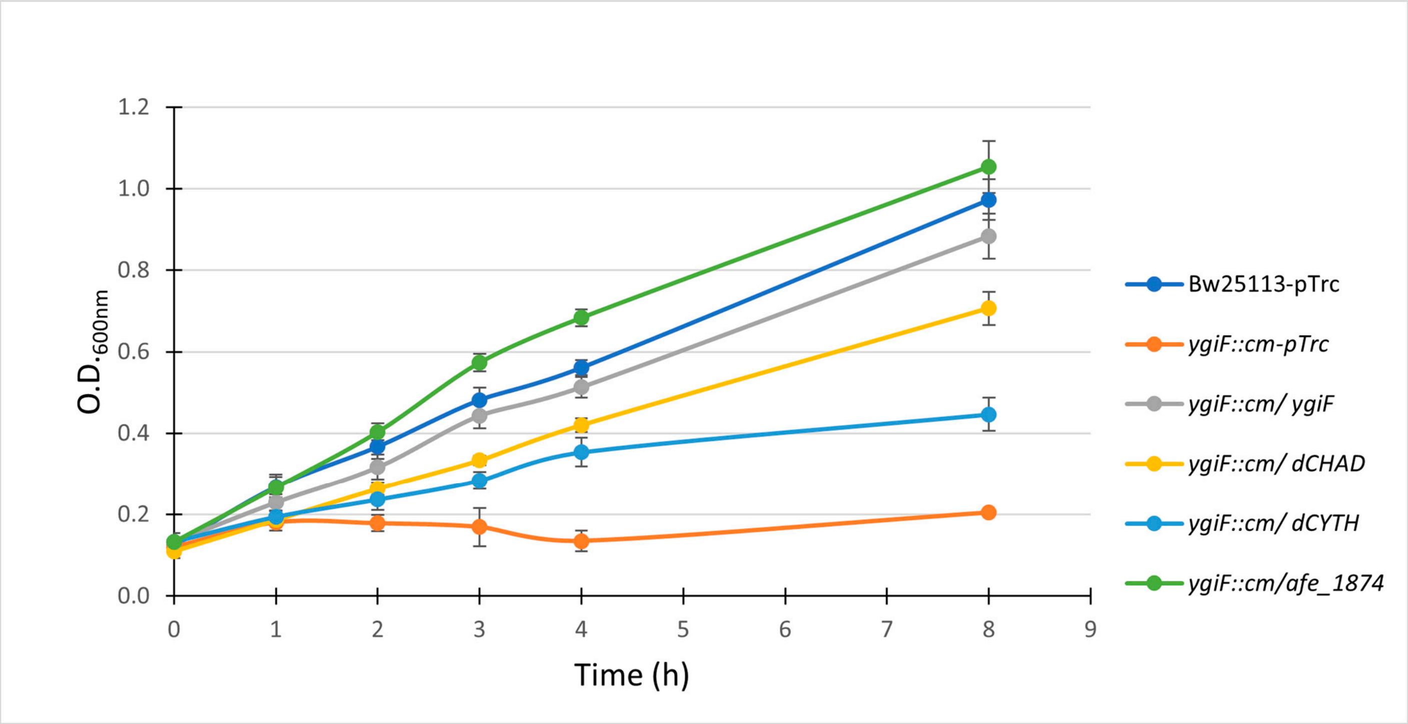

Figure S8. **Complemented strains restore wildtype phenotype**. Copper resistance of *E. coli* complemented strains. Cells were grown in LB medium supplemented with 1 mM IPTG in the absence of copper until they reached an $O.D._{600}$ of 0.1 At this point. Cells were then grown in LB medium supplemented with $CuSO_4$ to reach 3 mM final concentration. Finally, cultures were incubated at 37°C and optical density was measured in each indicated strain at the indicated times. Three biological replicas were obtained in each strain construction.

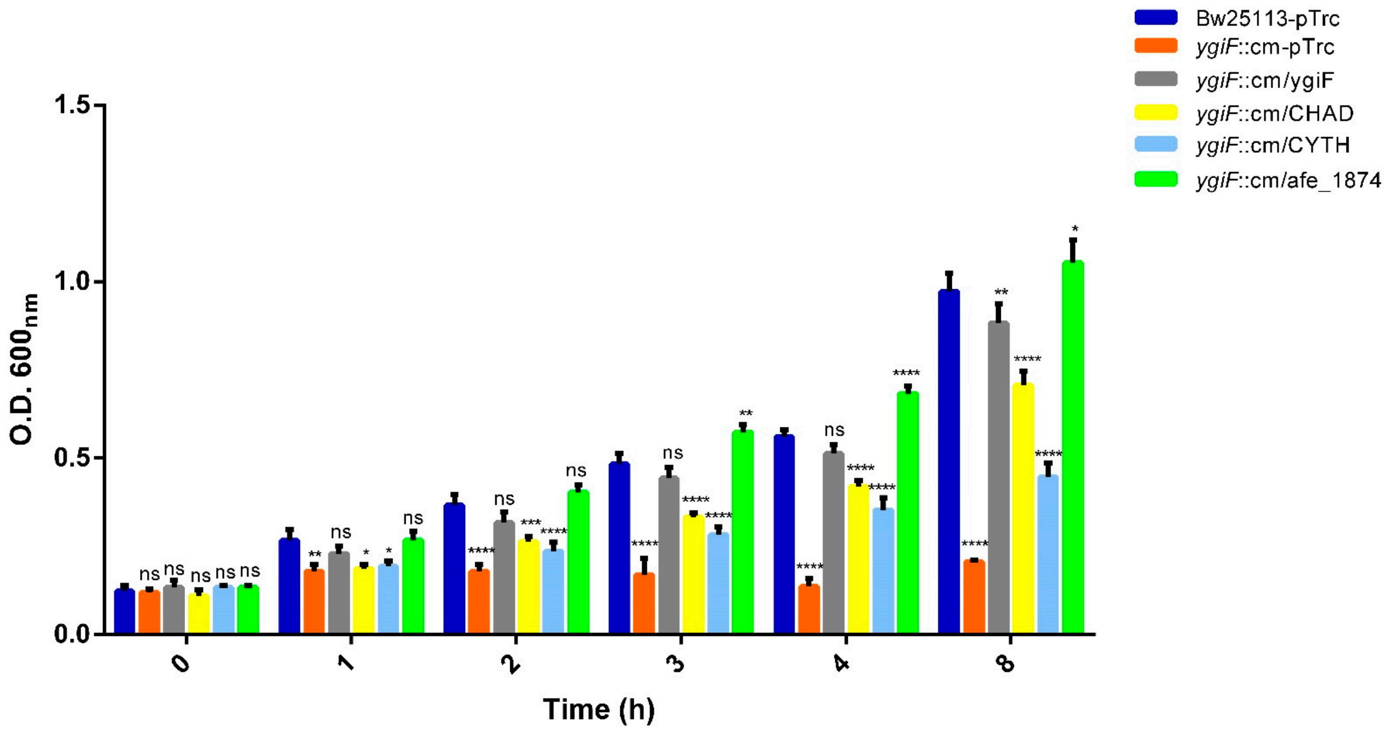

Figure S9. Statistical analysis of copper resistance in different *E. coli* mutants. Two-way ANOVA test was used, where **** indicates $p \leq 0.0001$, *** $p \leq 0.001$, ** $p \leq 0.01$, * $p \leq 0.05$ and ns not significant.

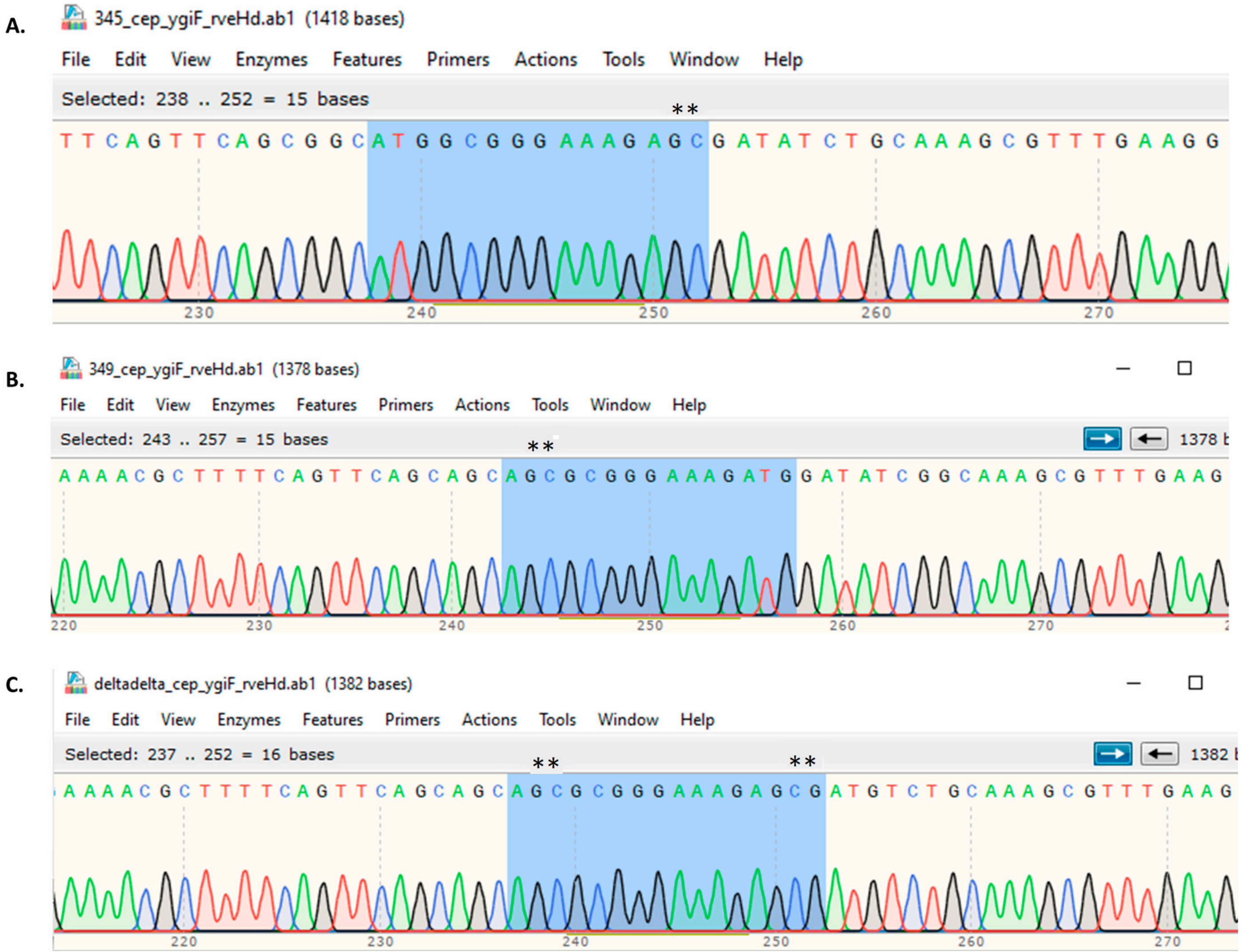

Figure S10. Part of the nucleotide sequences of mutated *ygiF* genes constructed in *E. coli*. A. *ygiF**H345A, **B.** *ygiF**H349A and **C.** *ygiF**H345,349A. The light blue rectangle indicates the amino acids "***HRLSH"*** region of interest. The asterisks indicate the mutated nucleotides. Note the "ATG " sequence codifies for a histidine that was replaced by "AGC" coding for an alanine in His345 (A), His349 (B) and both histidines in C.

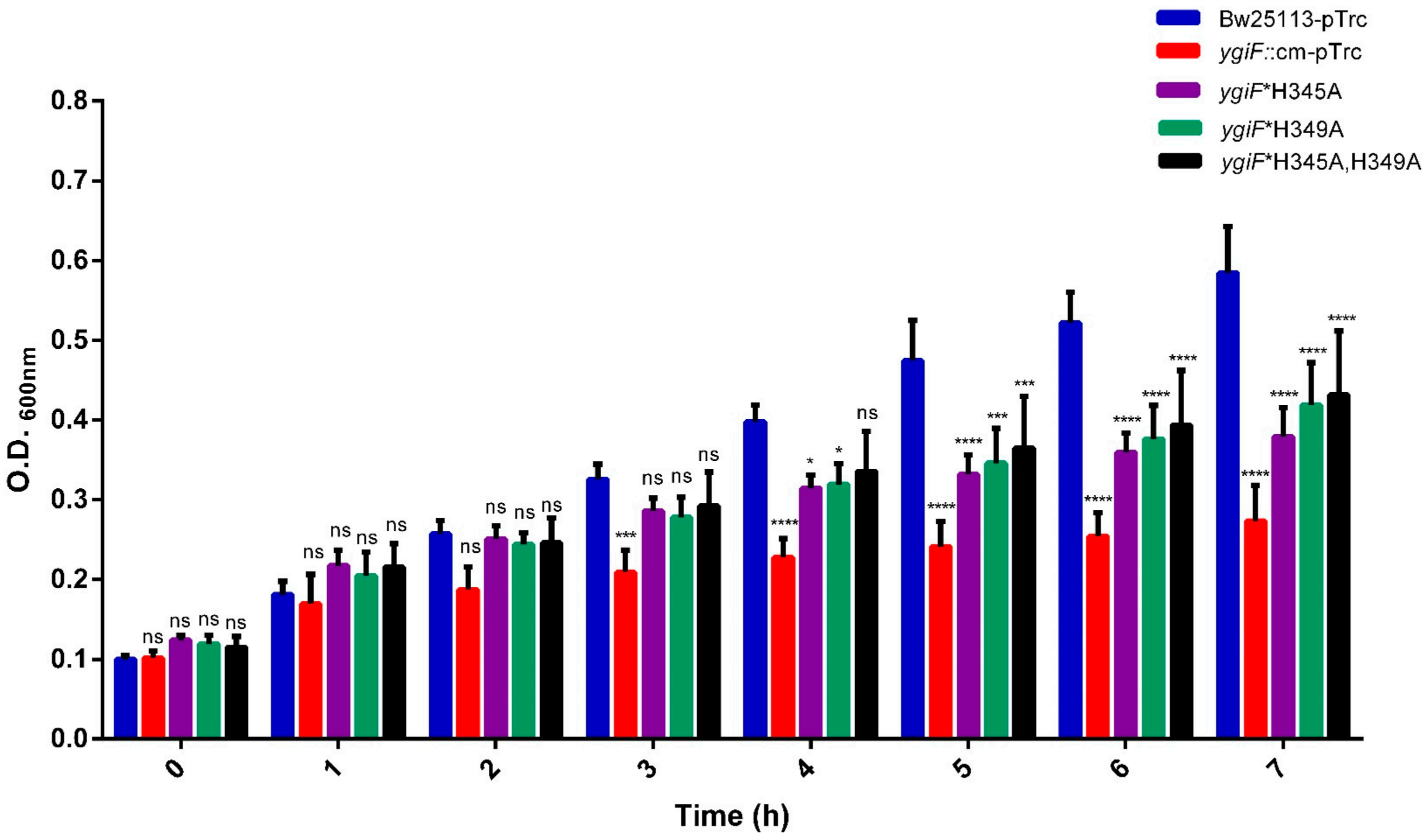

Figure S11. Statistical analysis of copper resistance in YgiF variant *E. coli* strains. Two-way ANOVA test was used, where **** indicates $p \leq 0.0001$, *** $p \leq 0.001$, ** $p \leq 0.01$, * $p \leq 0.05$ and ns not significant.
