# Supplementary figures and images for: Possible Role of CHAD Proteins in Copper Resistance
Source: Microorganisms. 2024 Feb 18;12(2):409. doi: 10.3390/microorganisms12020409 (PMC10892726; doi:10.3390/microorganisms12020409)

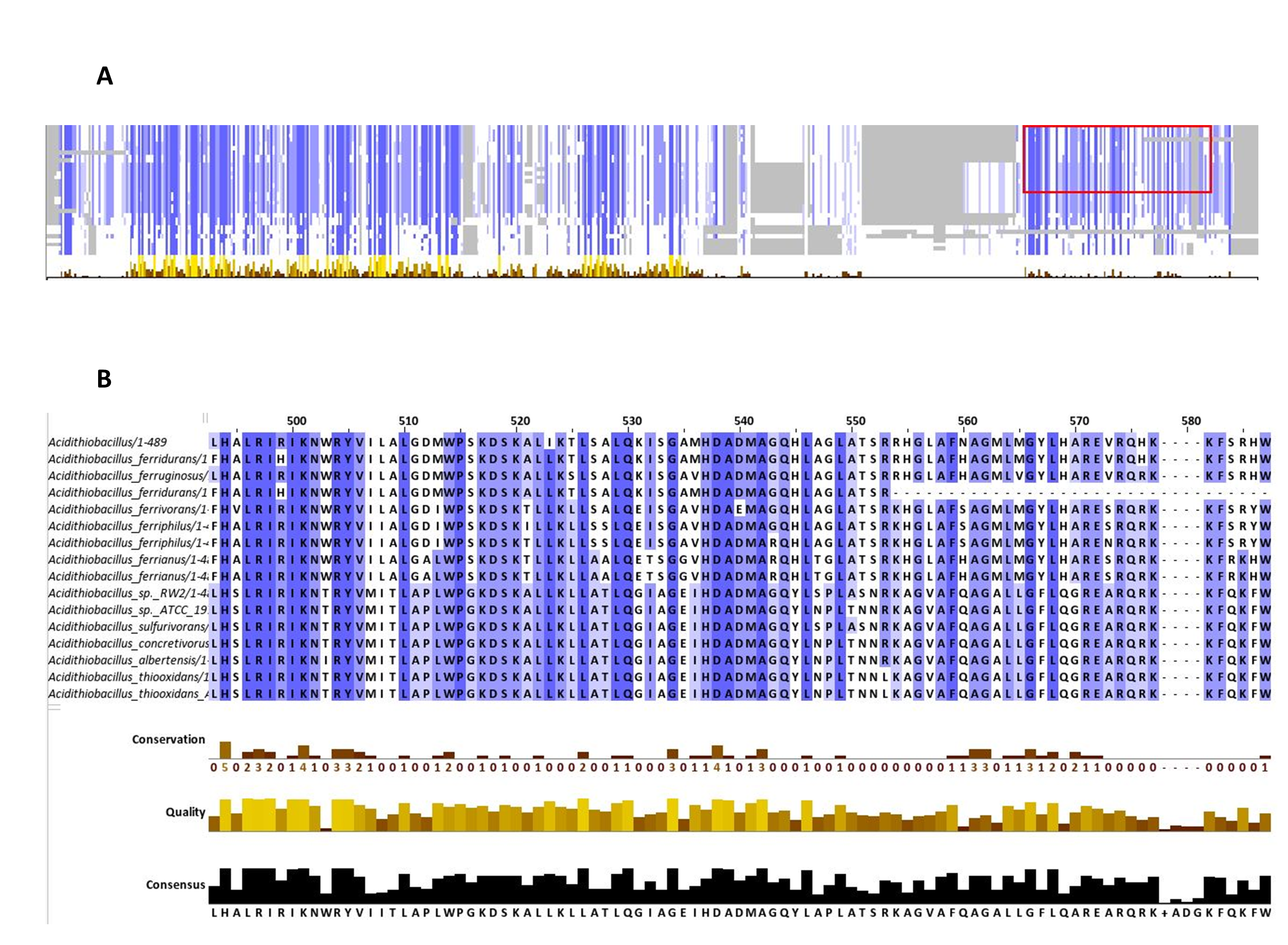

Supplement: Supplementary file 1 [file microorganisms-12-00409-s001.zip › Fig S1.tiff]

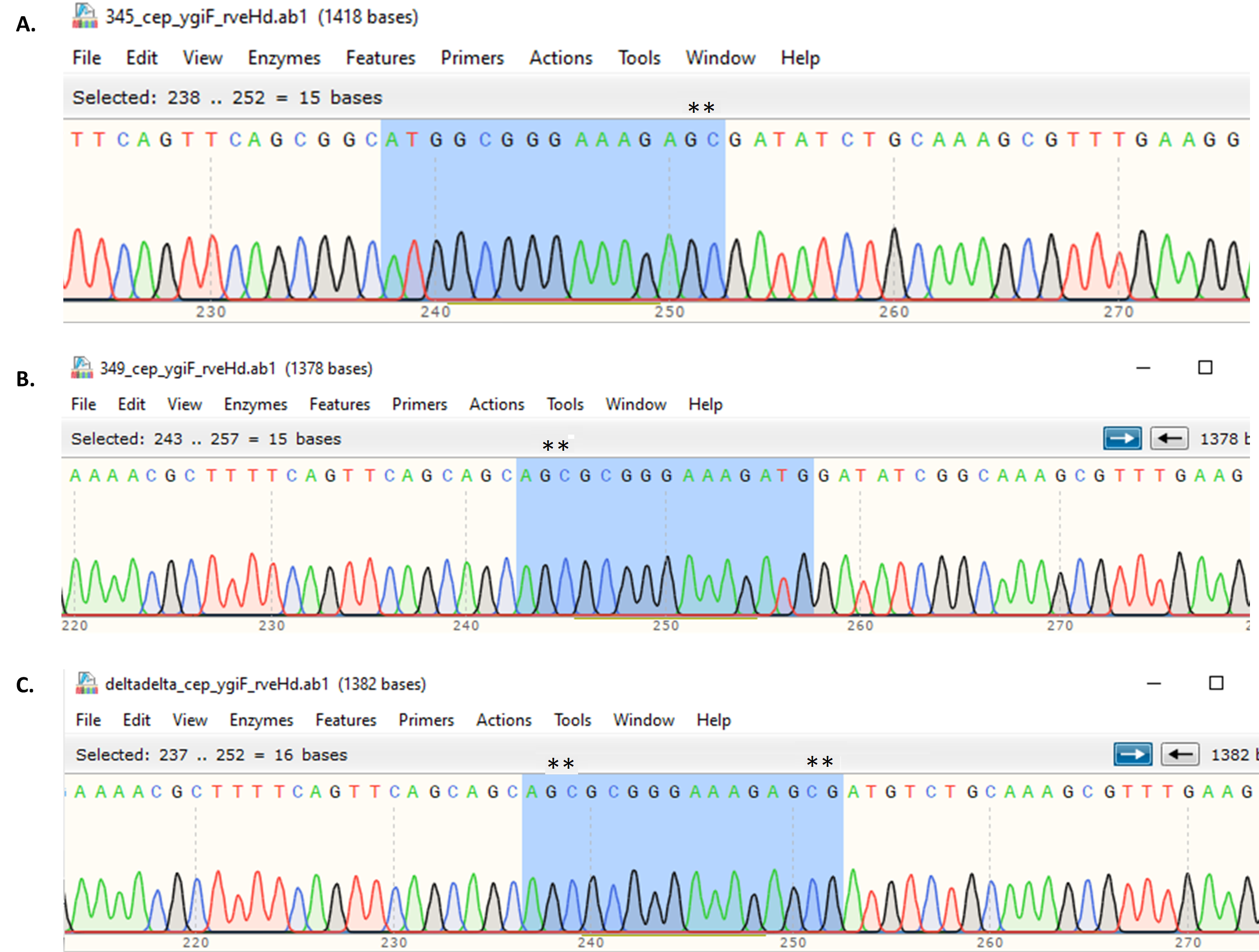

Supplement: Supplementary file 1 [file microorganisms-12-00409-s001.zip › Fig S10.tiff]

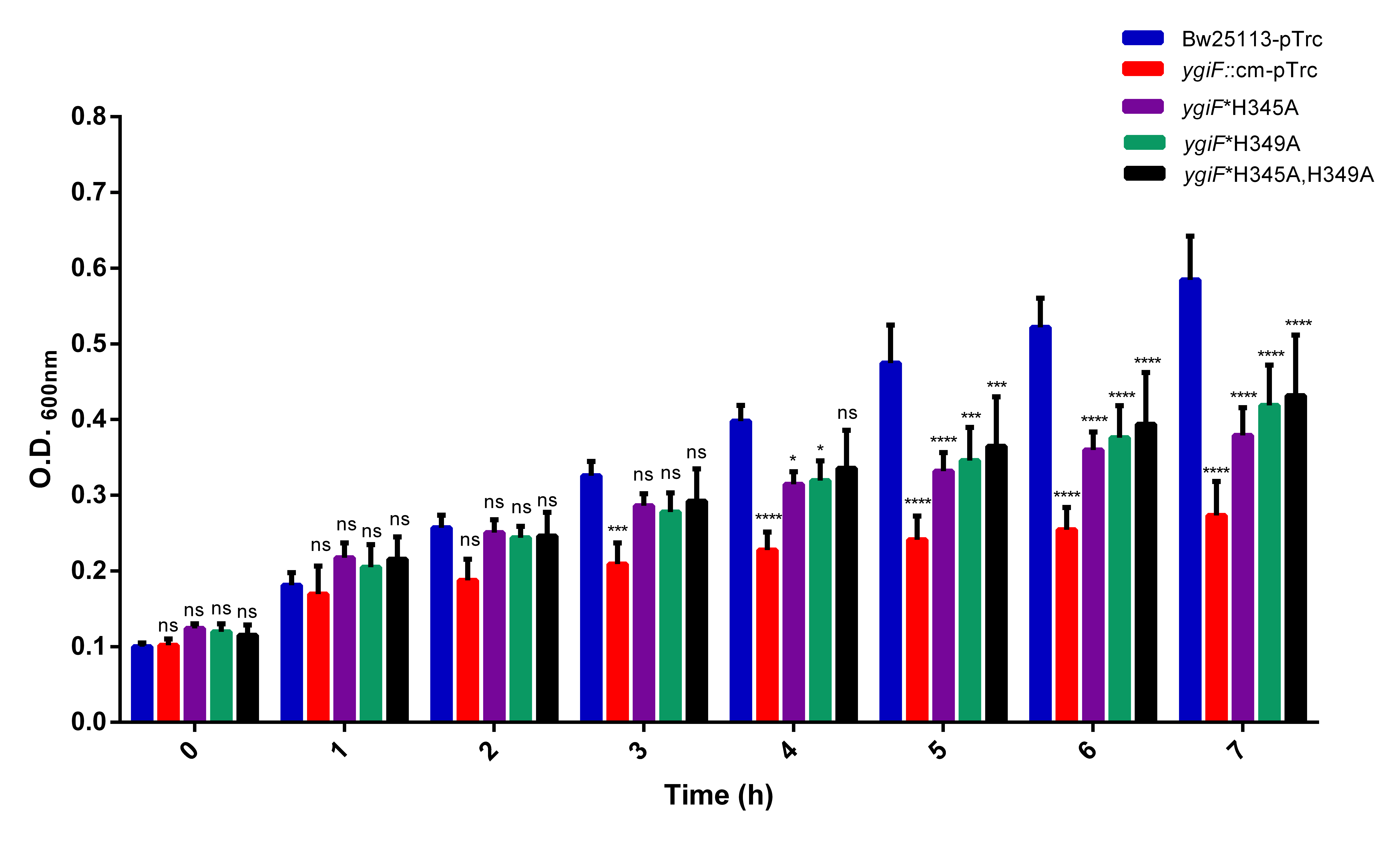

Supplement: Supplementary file 1 [file microorganisms-12-00409-s001.zip › Fig S11.tiff]

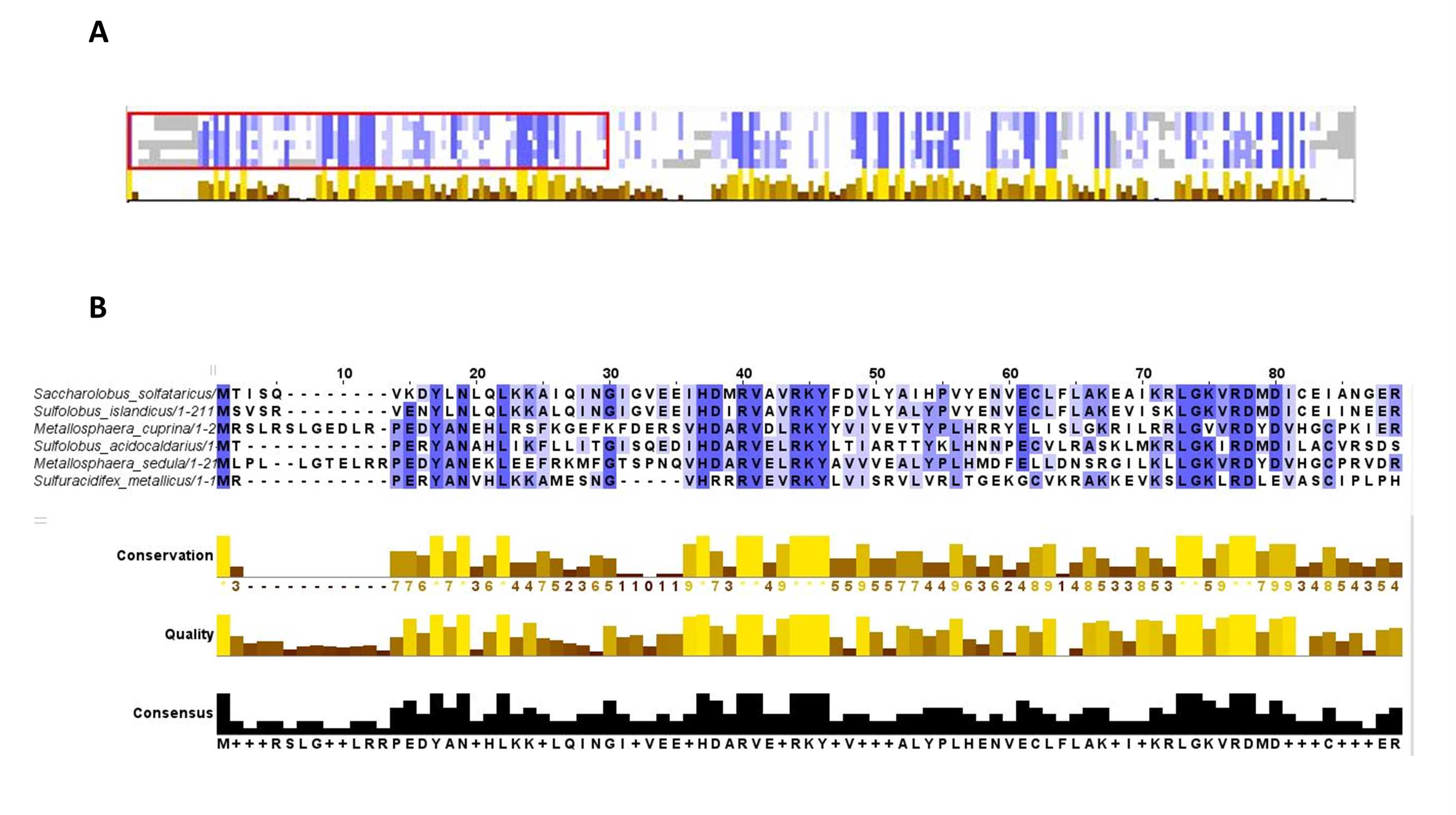

Supplement: Supplementary file 1 [file microorganisms-12-00409-s001.zip › Fig S2.tiff]

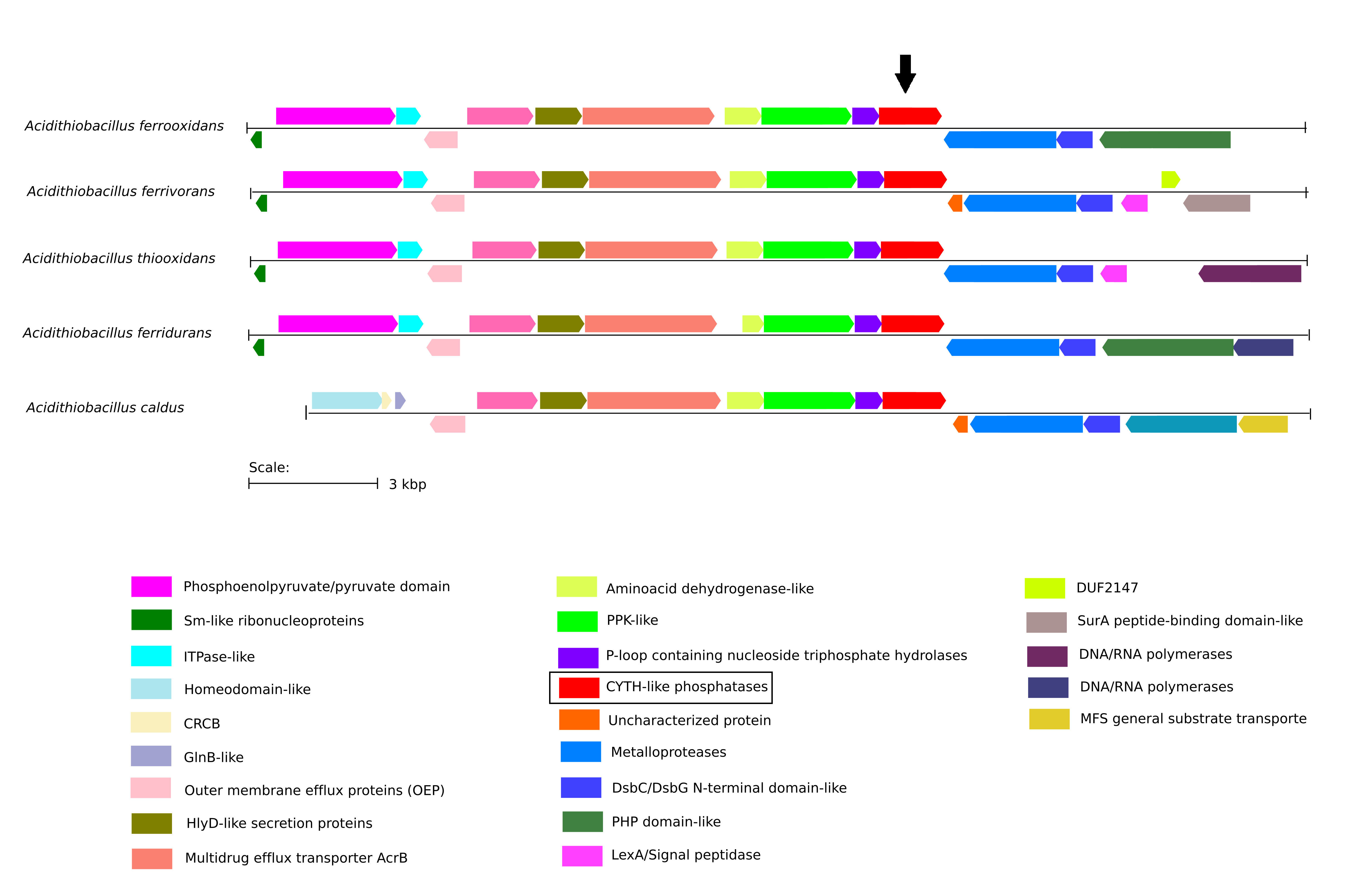

Supplement: Supplementary file 1 [file microorganisms-12-00409-s001.zip › Fig S3.tiff]

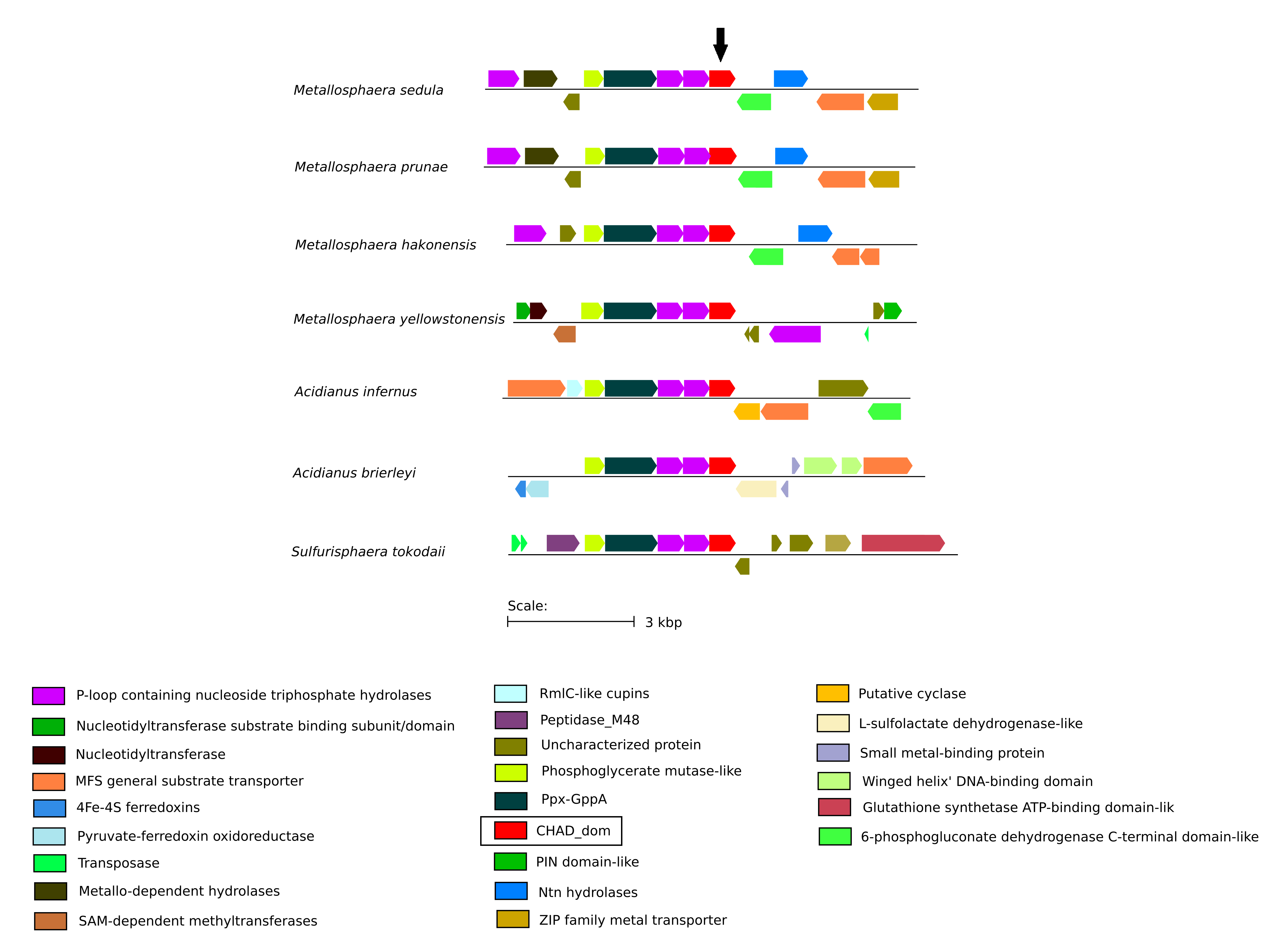

Supplement: Supplementary file 1 [file microorganisms-12-00409-s001.zip › Fig S4.tiff]

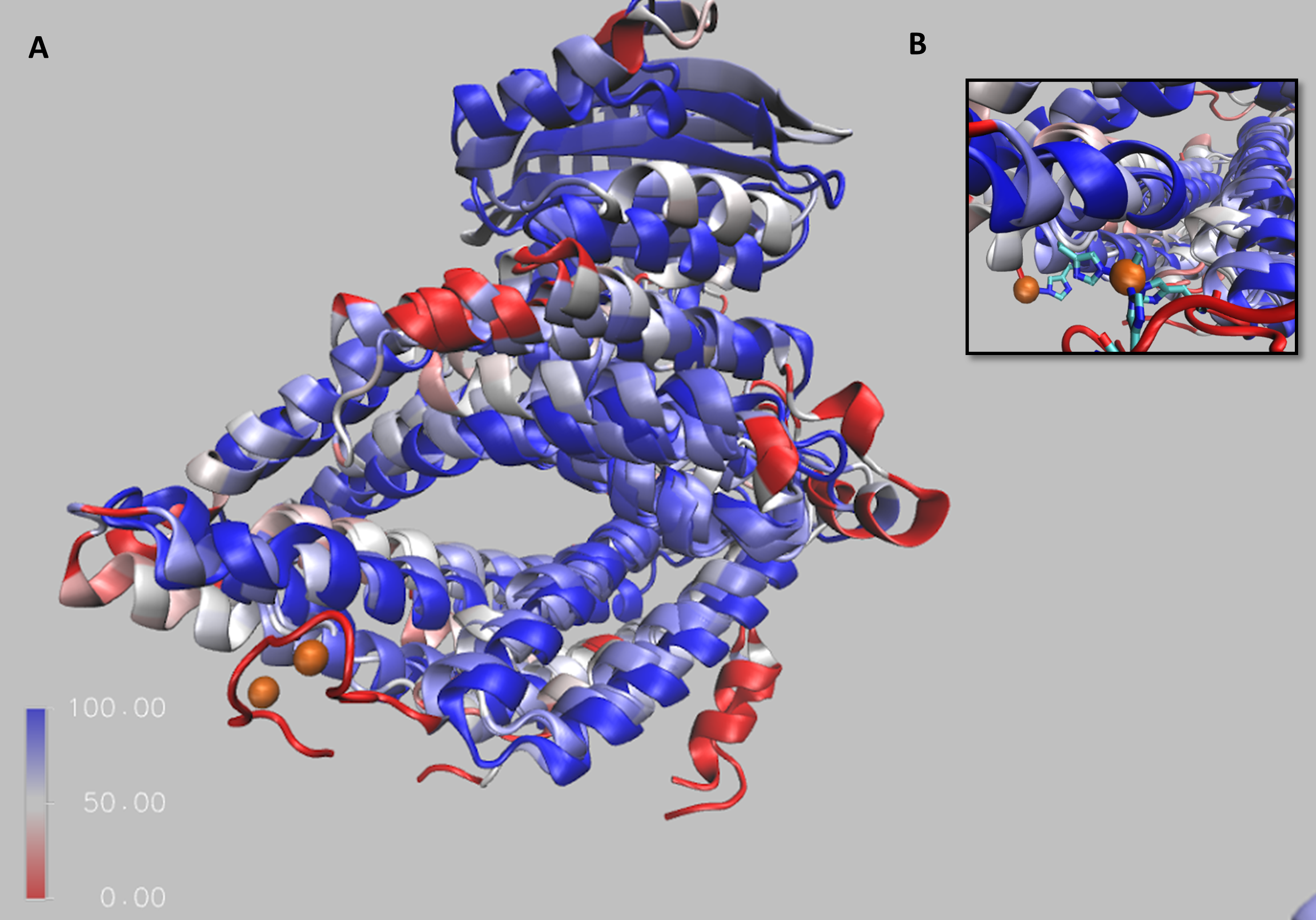

Supplement: Supplementary file 1 [file microorganisms-12-00409-s001.zip › Fig S5.tiff]

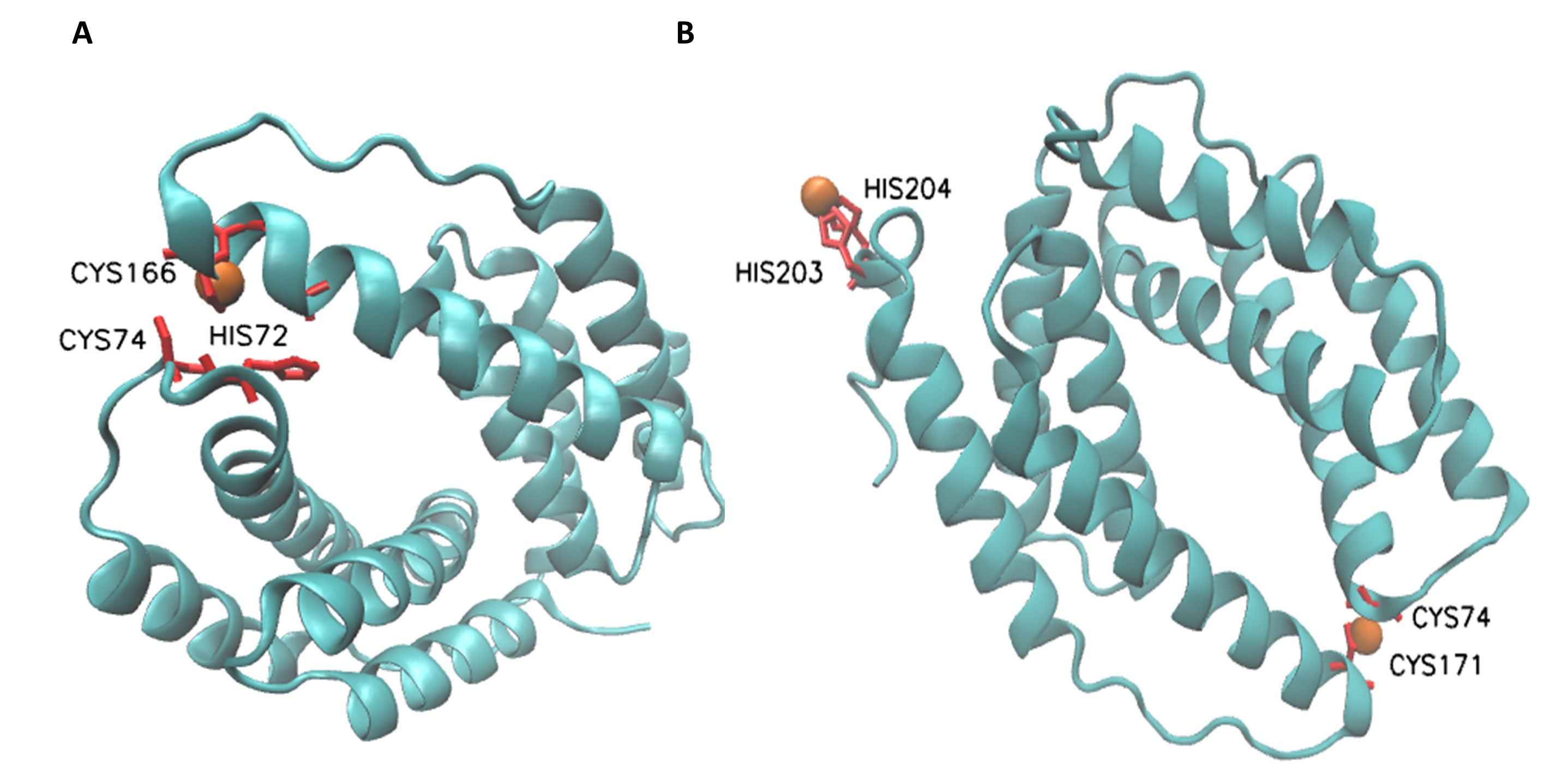

Supplement: Supplementary file 1 [file microorganisms-12-00409-s001.zip › Fig S6.tiff]

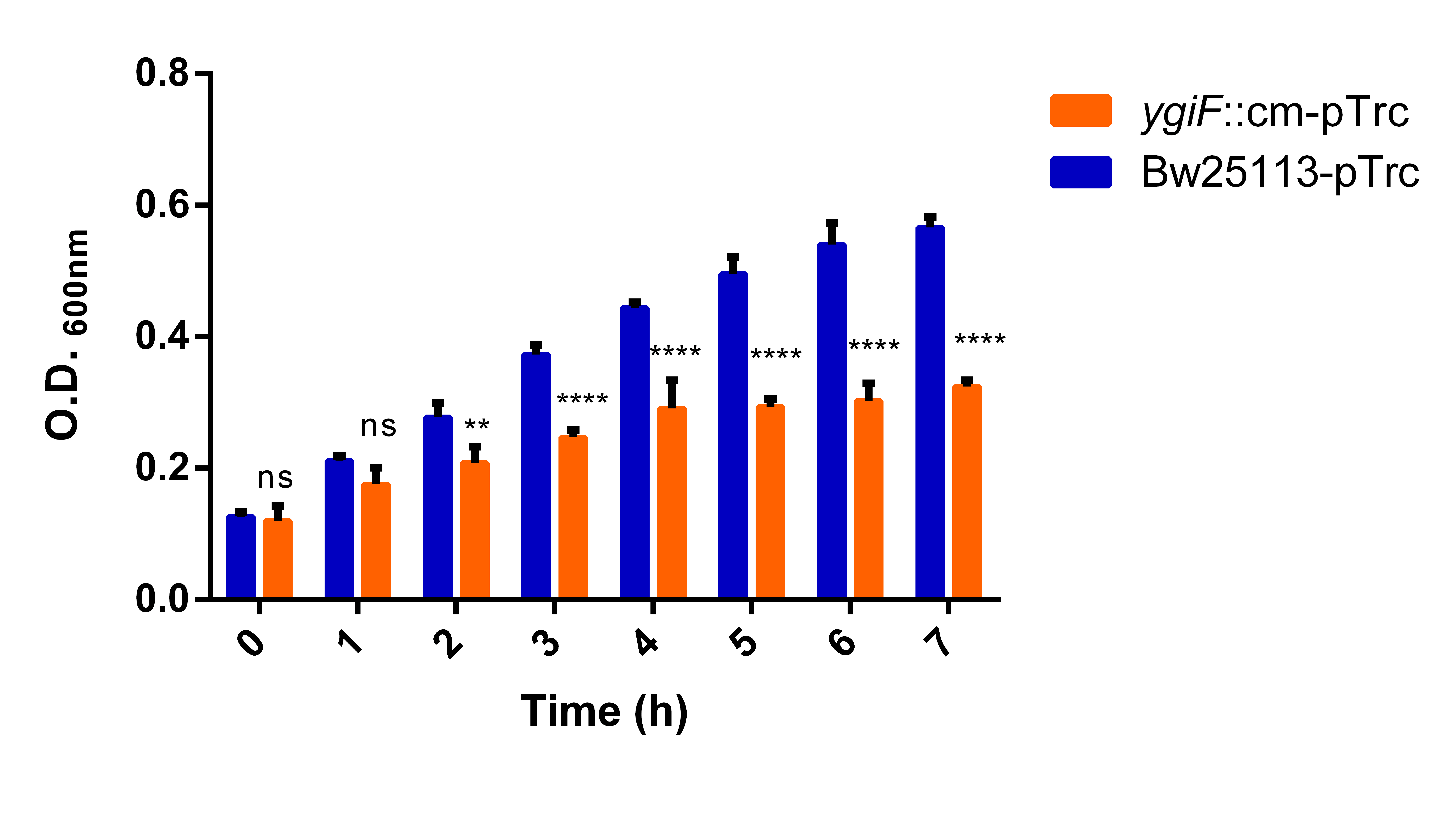

Supplement: Supplementary file 1 [file microorganisms-12-00409-s001.zip › Fig S7.tiff]

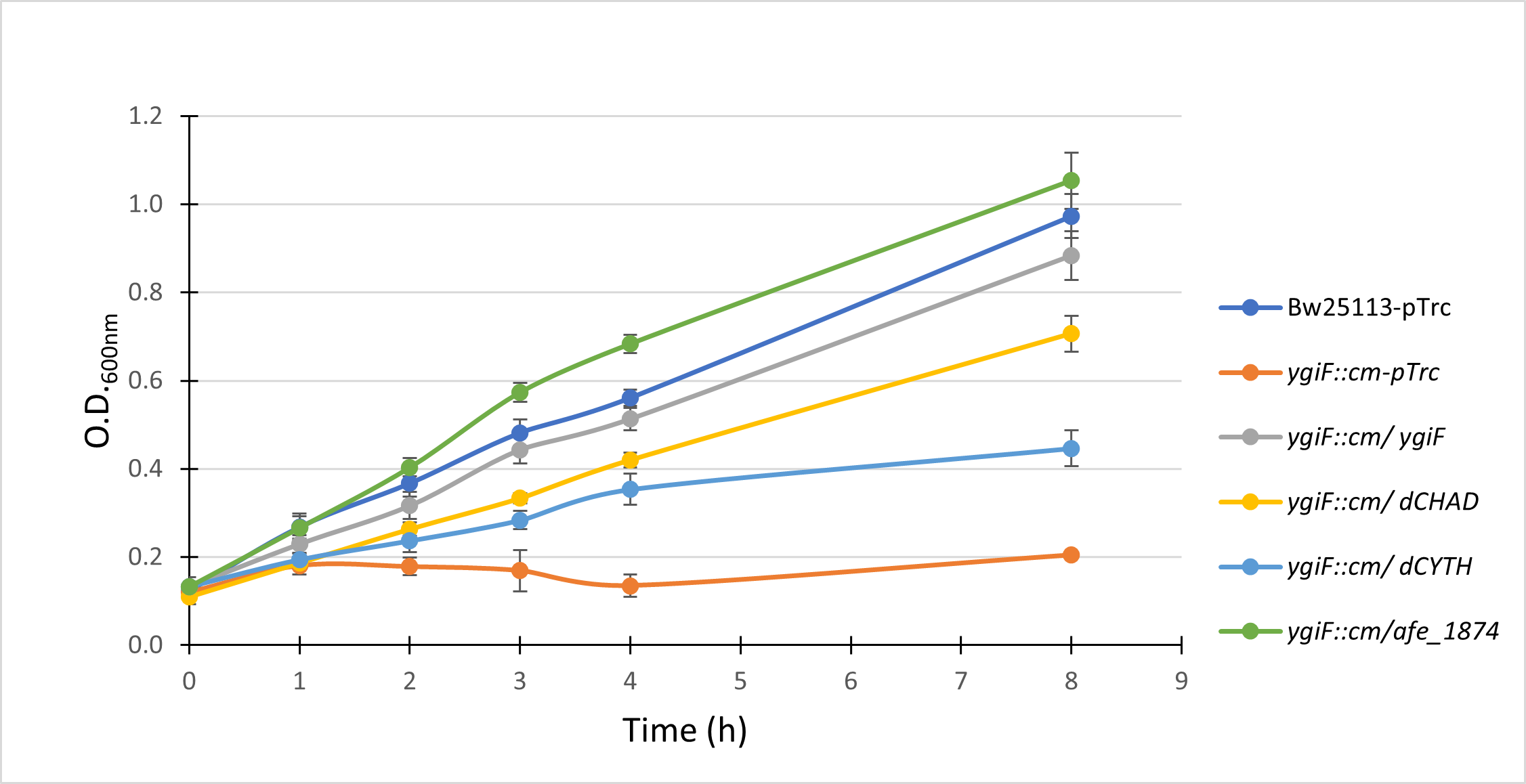

Supplement: Supplementary file 1 [file microorganisms-12-00409-s001.zip › Fig S8.tiff]

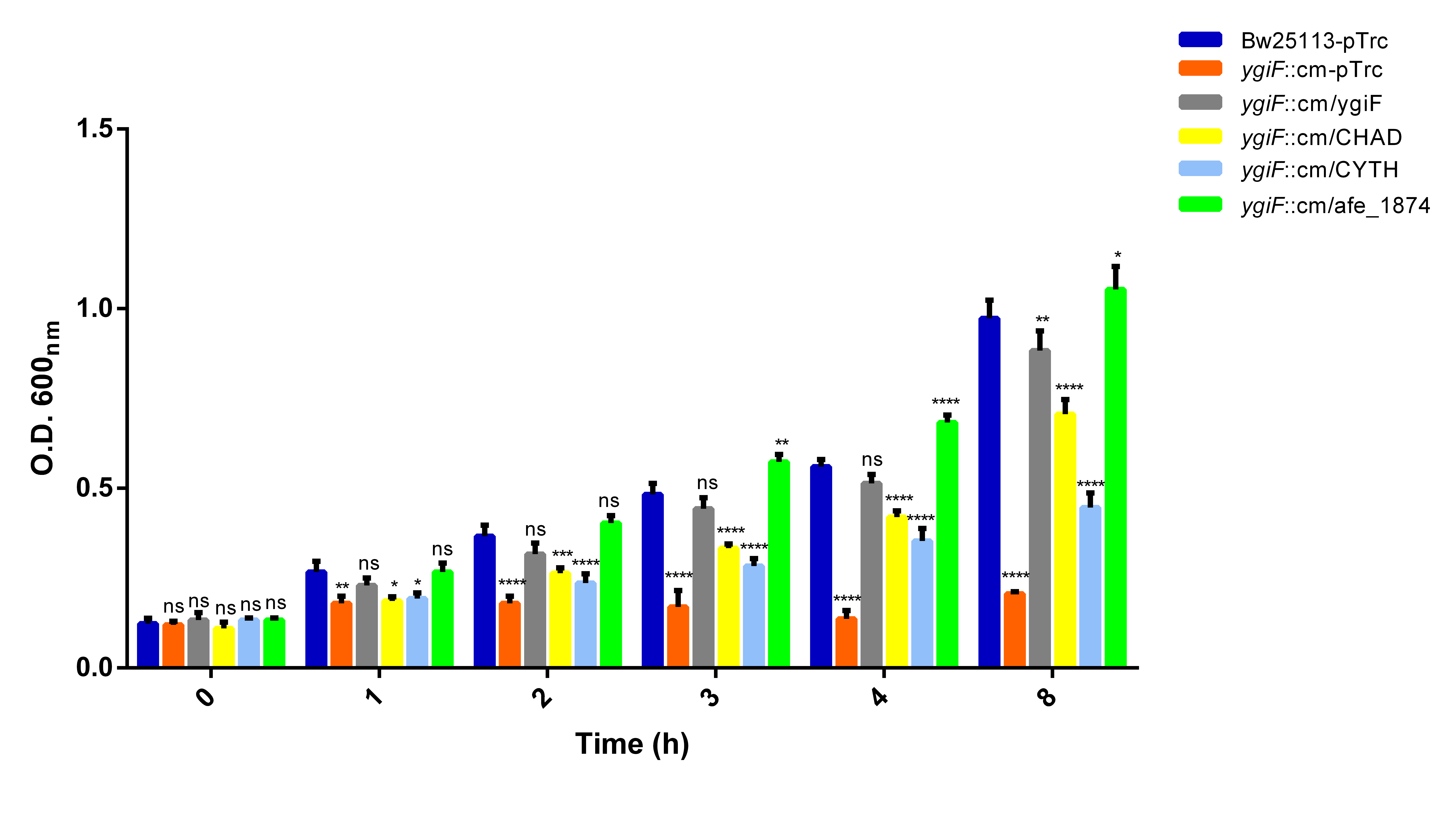

Supplement: Supplementary file 1 [file microorganisms-12-00409-s001.zip › Fig S9.tiff]
